# Does one plus one always equal two? Structural differences between nesfatin-1, -2, and nesfatin-1/2

**DOI:** 10.1186/s12964-022-00980-7

**Published:** 2022-10-24

**Authors:** Rafał Lenda, Michał Padjasek, Artur Krężel, Andrzej Ożyhar, Dominika Bystranowska

**Affiliations:** 1 Department of Biochemistry, Molecular Biology and Biotechnology, Faculty of Chemistry, Wrocław University of Science and Technology, Wybrzeże Wyspiańskiego 27, 50-370 Wrocław, Poland; 2 Department of Chemical Biology, Faculty of Biotechnology, University of Wrocław, Joliot-Curie 14a, 50-383 Wrocław, Poland

**Keywords:** Nucleobindin-2, Nesfatin-1, Nesfatin-2, Zinc, Intrinsically disordered protein, IDP, Metalloprotein, Hormone, Neuropeptide, Satiety molecule

## Abstract

Nesfatin-1 and -2 are produced from a reaction in which the N-terminus of human Nucleobindin-2 undergoes proteolytical processing. To date, Nucleobindin-2 and/or nesfatin-1 have only been shown to act as peptide hormones. On the other hand, the purpose of nesfatin-2 remains unknown. Since Nucleobindin-2/nesfatin-1 is thought impact the control of a wide range of physiological processes, including energy homeostasis, neurodegenerative processes and carcinogenesis, its ligands/interactions deserve special studies and attention. However, there are no reports about the molecular properties of the proteolytical products of human Nucleobindin-2 in the literature. Hence, this study aimed to analyze the effect of Zn(II) and Ca(II) on human nesfatin-1, -2, and -1/2 structures. Herein, we report that human nesfatin-1 is a member of the intrinsically disordered protein family, as indicated by circular dichroism and analytical ultracentrifugation experiments. In contrast, we found that the human nesfatin-2 and nesfatin-1/2 structures were globular with intrinsically disordered regions. Under Zn(II) treatment, we observed concentration-dependent structurization and compaction of intrinsically disordered nesfatin-1 and its propensity for oligomerization, as well as destabilization of both nesfatin-2 and nesfatin-1/2. Furthermore, dissociation constants for Zn(II) binding by nesfatin-1, nesfatin-2, and nesfatin-1/2 were also reported. Moreover, structurally distinct nesfatin-1 and -2 seem to be interdependent when linked together, as indicated by the observed molecular properties of nesfatin-1/2, which in turn are not a simple sum of the properties exhibited by the former peptides. Thus, herein, we shed new light on the molecular behavior of human nesfatins, which might help to elucidate the complex function of those peptides.

**Video abstract**

**Supplementary Information:**

The online version contains supplementary material available at 10.1186/s12964-022-00980-7.

## Introduction

**Fig. 1:**
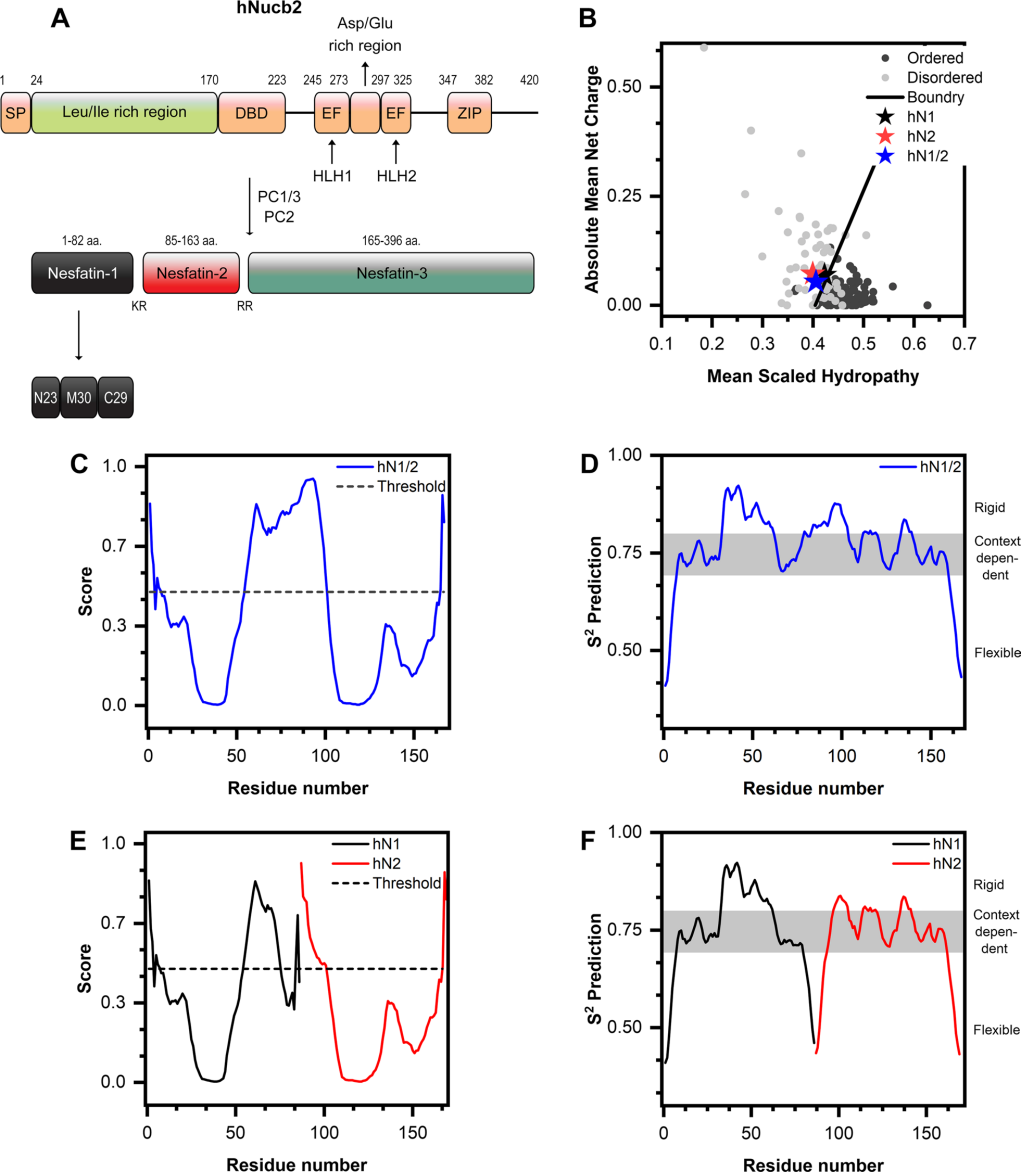
Proteolytical processing and potential intrinsic disorder regions of human nesfatins. (**A**) Schematic representation of the hNucb2 structure and products of its proteolytical processing. (**B**) Charge-hydropathy analysis. The dark gray circles represent the set of 105 completely ordered proteins, and the light gray circles represent the set of 54 completely disordered proteins. The stars represent hN1 (black), hN2 (red) and hN1/2 (blue) proteins. The solid black line is a boundary between the ordered and the unordered region. (**C**) Prediction of hN1/2, (**E**) hN1 and hN2 IDRs with the PONDR algorithm. Scores ≥ 0.5 indicate putative disordered regions. (**D**) The flexibility of the hN1/2, (**F**) hN1 and hN2 backbones calculated with the DynaMine algorithm. The S^2^ parameter is a measure of the N–H bond rotational freedom. Below the 0.69 threshold, the region is predicted to be flexible, and above the 0.8 value, the residues are predicted to be rigid. The zone between those thresholds is context dependent

Nucleobindin-2 (Nucb2) and/or nesfatin-1 were first discovered as central and peripheral hormones that are engaged in the regulation of energy homeostasis [1]. Since then, numerous reports have identified their involvement in the tissue-specific regulation of carcinogenesis [2–4], circadian rhythm, inflammation [5, 6], obesity [7], the pathogenesis of psychoneuronal disorders [8, 9], and more. On the other hand, there are still few reports about the molecular properties of nesfatins and Nucb2 and their relationship to their function. Human nesfatin-1 (hN1) and -2 (hN2) are formed in vivo by the action of specific pro-hormone convertases 1/3 (PC1/3) and 2 (PC2) on human Nucb2 (hNucb2, Fig. 1A) [10]. Full-length hNucb2 is composed of 396 amino acid residues (aa.) and preceded by a 24-aa. signal peptide (Fig. 1A). hNucb2 is characterized by a multidomain structure (Fig. 1A). Beginning from the N-terminus, the structure contains a Leu/Ile rich region followed by the DNA-binding domain (DBD), two EF-hand domains flanking the Asp/Glu rich region, and a leucine zipper (ZIP) motif on the C-terminus [11]. The structure of hN1 can be divided into the following segments (Fig. 1A): the N-terminal fragment (N23), middle fragment (M30), and C-terminal fragment (C29). The anorexigenic mode of action of nesfatin-1 is believed to be associated with the M30 fragment [12].

The multidomain nature of Nucb2 is partially reflected by its wide spectrum of physiological functions, as discussed below. The recently described propensity of *Gallus gallus* (ggNucb2) and hNucb2 homologs for intrinsic disorder [13] seems to complete this picture, since multifunctionality is a characteristic feature of intrinsically disordered proteins (IDPs) and proteins that contain intrinsically disordered regions (IDRs) [13, 14]. IDPs and IDRs lack a defined tertiary structure and thus escape the classical structure–function paradigm; thus, the function and specificity of the protein is the outcome of its ordered structure [15]. Instead, IDPs and IDRs exhibit a highly dynamic molecular structure that allows them to display a broad spectrum of functions, and these structures can be divided into the following classes: entropic chains, display sites, chaperones, effectors, assemblers, and scavengers [14, 15]. Another characteristic feature of IDPs and IDRs is their frequent disorder-to-order transition upon protein partner/ligand binding and their strong susceptibility to posttranslational modifications (PTMs) that further regulate the complex signaling and interaction networks of IDPs [14–16]. Skorupska et al. [13] revealed that Nucb2s have a mosaic structure, in which N-terminal fragments were shown to comprised an alternating ordered and disordered fragments [13]. N-terminal fragments of Nucb2s were also shown by Bystranowska et al. [17] to bind Zn(II), which further augments the biological importance of Nucb2. This knowledge leads to questions regarding the molecular properties of nesfatin-1 (N1) and -2 (N2) after they are proteolytically released from Nucb2 by prohormone convertases (PCs) and whether they retain the mosaic structure and/or Zn(II) sensing abilities of Nucb2. However, to the best of our knowledge, there are no reports on the molecular properties of N1, N2, and N1 coupled with N2 in a head-to-tail manner (nesfatin-1/2, N1/2, encompassing the N-terminal half of the Nucb2 molecule) to date. Moreover, in addition to N1, the function of N2 and N1/2, which are in vivo products of PC action, is still elusive; nonetheless, they might be critical for the regulation of N1 activity and may be physiologically significant.

Hence, in this paper, we describe the molecular properties of hN1, hN2, and hN1/2 and the effect of Zn(II) and Ca(II) on their structure. Our results indicate that hN1 in its apo form displays a highly disordered structure that, upon Zn(II) treatment, undergoes disorder-to-order transition accompanied by dimerization of the peptide and formation of the hydrophobic core. We also observed that Zn(II) is bound by ordered hN2 and hN1/2; however, despite the tighter binding by hN1/2, this interaction was strongly structure-destabilizing. The ordered structure of hN2 and hN1/2 peptides along with the distinct structure of the free hN1 fragment imply that in vivo proteolytic processing of hNucb2 by PCs and further processing of hN1/2 may act as an activation mechanism, which in turn might assign the hN2 fragment a structural, yet unclear role. Thus, the results presented in this paper provide new insight into the molecular properties of hN1, hN2, and hN1/2. The structures of hN1 and hN2 appear entirely dissimilar; however, the peptides seem to be structurally interdependent, as shown by the properties of hN1/2, which in turn might have extensive physiological implications that undoubtedly necessitate further investigation.

## Materials and methods

### Chemicals

#### Buffers

Buffer A1 (50 mM NaH_2_PO_4_ × 2H_2_O, pH 7.0; 300 mM NaCl), Buffer A2 (50 mM NaH_2_PO_4_ × 2H_2_O; 300 mM NaCl; 200 mM imidazole, pH 7.0), Buffer B (20 mM Tris–HCl, pH 7.5; 150 mM NaCl). Buffer C (20 mM MES, pH 6.5; 150 mM NaCl). All buffers were prepared at ambient temperature and filtered through a 0.22 μm filter.

#### Reagents

DNase I, RNase I, imidazole, EDTA, chloramphenicol (C), phenylmethylsulfonyl fluoride (PMSF), 2-carboxy-2′-hydroxy-5′-sulfoformazylbenzene monosodium salt (Zincon, ZI) were purchased from Sigma Aldrich. Carbenicillin (R) was purchased from Roth. All remaining chemicals were of analytical grade and are commercially available.

#### Primers

hN1F (GCGCGAGCTCGTGCCGATTGATATCGATAAA), hN1R (GCGCAAGCTTCTACAGTTCATCCAGTTTGGTAC), hN2F (GCGCGAGCTCCAAGAAGTTGGTCGTCTGC), hN2R (GCGCAAGCTTCTACTCGTGTTCTTTCATCATTTC).

#### Resins and columns

PD10 desalting and Superdex 75 Increase 10/300 GL columns were purchased from GE Healthcare. Ni–NTA agarose resin was purchased from Qiagen.

### In silico molecular analysis

Prediction of intrinsically disordered regions (IDRs) of hN1, hN2, and hN1/2 was based on their amino acid sequence and the following disorder prediction tools: PONDR-VL-XT [18, 19] (available at http://pondr.com). The backbone molecular dynamics of proteins was assessed with the DynaMine tool [20, 21] (available at http://dynamine.ibsquare.be/).

### Preparation of recombinant vectors

Sequences of hN1, hN2 and hN1/2 were amplified via polymerase chain reaction (PCR) with primers that introduced SacI and BamHI restriction sites. The template was a modified recombinant pQE-80L (Qiagen) vector containing cDNA of hNucb2, which was prepared by our team previously [13]. Amplified Nucb2 cDNA fragments along with the pQE-80L vector were double-digested (2.5 h, 37 °C, 400 rpm) with SacI and BamHI endonucleases (Thermo Scientific). Then, the fragments were ligated (3 h, 37 °C, 500 rpm) with T4 DNA ligase (Thermo Scientific) into the pQE-80L vector. The obtained constructs were validated by Sanger sequencing (Genomed S.A.). The N-terminal sequence of proteins contained 6 × His tag followed by the Human rhinovirus 3C (HRV3C) protease cleavage site that was derived from the modified pQE-80L vector.

### Expression and purification of recombinant proteins

Bl21 (DE3) pLysS *E. coli* competent cells (Thermo Scientific) were transformed with 4 ng of each construct and incubated overnight on LB (50 μg/ml of R; 35 μg/ml of C) agar. The chosen transformants were transferred to TB (R + C) medium and incubated for 8 h at 37 °C and 200 rpm. Then, 30 ml (6% v/v) of the above inoculum was added to 0.5 l of TB (R + C) medium and incubated for 5 h at 29 °C and 200 rpm until an optical density of 0.7–0.8 at 600 nm was reached. Subsequently, IPTG was added to the culture medium to a final concentration of 0.25 mM, and the culture was incubated for the next 3 h at 29 °C and 200 rpm. The cells were harvested by centrifugation for 8 min at 4 °C and 5,500 g, and the pellet was suspended in 12 ml of A1 buffer supplemented with 20 μg/ml PMSF. All extracts were stored at -80 °C before use. Then, the extracts were thawed on ice, and PMSF (20 μg/ml), DNase I (10 μg/ml), and RNase I (10 μg/ml) were added. Cell lysis was conducted by sonication on ice followed by incubation for 1 h at 4 °C and 10 rpm on a vertical shaker to remove nucleic acids. The cell lysates were centrifuged (1 h, 4 °C, 18,000 g), and the supernatant was collected, followed by the subsequent addition of 2 ml (50%) of preequilibrated (buffer A1) Ni–NTA resin. The supernatant was then incubated for 30 min at 4 °C and 10 rpm. The supernatant was transferred onto an empty Tricorn column, which was subsequently connected to an Äkta Explorer (GE Healthcare) system. The resin was washed with 20 bed volumes of buffer A1. Next, contaminant proteins were eluted with 10 bed volumes with buffer A1 supplemented with 35 mM imidazole (hN1) or 20 mM imidazole (hN2, hN1/2). Finally, the proteins of interest were eluted with 10 bed volumes of buffer A2, and the chosen fractions were pooled and desalted to buffer A1 on a PD10 desalting column according to the manufacturer’s protocol. Then, HRV3C protease (Sino Biological) was added at a 1:100 w/w ratio, and the digestion solution was incubated overnight at 4 °C and 10 rpm. The above solution was then incubated with 0.6 ml of preequilibrated Ni–NTA resin for 30 min at 4 °C and 10 rpm and loaded onto the column. Flow-through was collected and concentrated on Amicon Ultra-4 centrifugal filter units (Millipore) to a 500-μl volume. Proteins of interest were further purified by size exclusion chromatography (SEC) on a Superdex 75 Increase 10/300 GL column connected to the Äkta Avant chromatography system (GE Healthcare). Protein separation was monitored by the A_280_ measurement, and the concentration was estimated with the following extinction coefficients: 4470 1/(M × cm) (hN1), 2980 1/(M × cm) (hN2) and 7450 1/(M × cm) (hN1/2). The chosen fractions were pooled, aliquoted, and stored at -80 °C until future use. The chemical purity and identity of the proteins were verified by SDS–PAGE analysis according to Laemmli [22] and mass spectrometry experiments, as shown in Additional file 1: Figs. S1 and S2, respectively.

### CD spectroscopy

Circular dichroism (CD) spectra were acquired with a Jasco J-815 CD-spectropolarimeter equipped with a Peltier-type temperature controller and quartz cuvettes with a 0.1 cm optical path length. The chosen spectral accumulation parameters were a scanning rate of 50 nm/min, a 1 nm bandwidth, and a wavelength range of 190–260 nm. The protein concentration was 0.15 mg/ml in each sample. Each spectrum was obtained at 20 °C with 3 accumulations per sample. The proteins were suspended in buffer B or buffer B supplemented with either CaCl_2_ (10 mM), EDTA (5 mM) or ZnCl_2_ (20–500 μM). The CD spectra of an appropriate buffer were subtracted from each protein spectrum, and the CD data were converted to the mean residue ellipticity. Raw data were further smoothed with the Savitzky–Golay filter (15 points, polynomial order 3). The smoothed data from the replicates were averaged. Deconvolution of individual CD spectra was performed with the CDPro package using the CONTINLL algorithm and SDP48 (hN1) or SP43 (hN2, hN1/2) set as a reference [23–25]. CD data of proteins titrated with Zn(II) ions were processed as performed above, and the fractional saturation and free Zn(II) concentration were calculated according to Eqs. (1) and (2), respectively. The obtained data were fitted against the Hill model with OriginPro 2018 software with the Hill coefficient set to 2 (hN1) or left unrestricted (hN2, hN1/2).

1 $$r = \frac{{\left| {\theta_{i, \;208/222} - \theta_{\min } } \right|}}{{\left| {\theta_{\max } - \theta_{\min } } \right|}}$$

where

$\theta_{i,\;208/222}$ is the mean residue ellipticity at 208 or 222 nm and at the i-th concentration of Zn(II), and $\theta_{{{\text{min}}}} ,\theta_{{{\text{max}}}}$ is the minimal and maximal observed mean residue ellipticity (MRE) at 208/222 nm.

2 $$\left[ {\text{Zn(II)}} \right]_{{{\text{free}}}} = \left[ {\text{Zn(II)}} \right]_{{\text{t}}} - r \times \left[ {\text{P}} \right]_{t}$$

where

$\left[ {\text{Zn(II)}} \right]_{{\text{t}}}$ is the total concentration of Zn(II),

$r$ is the fractional saturation,

$\left[ {\text{P}} \right]_{t}$ is the total concentration of protein.

### Analytical ultracentrifugation

Sedimentation-velocity analytical ultracentrifugation (SV-AUC) was conducted on a Beckmann Coulter Proteome-Lab XL-I ultracentrifuge (software version 6.0, Beckmann Coulter Inc.) endowed with an An-60Ti rotor. Protein solutions were prepared with two sets of concentrations, and the concentrations for hN1/2 were 1.85, 1.32, 0.92 mg/ml. For hN1 and hN2, the concentrations were 1.3, 1.0, 0.7 mg/ml. The protein samples were suspended in buffer B supplemented with either CaCl_2_ (10 mM), EDTA (5 mM) or ZnCl_2_ (50, 300, and 500 μM for hN1 and 50 μM for hN2 and hN1/2). The changes in tertiary and/or quaternary structures upon interaction of free hN1 and hN2 were detected by centrifuging the solution of the above peptides in the w/w ratio of 1:1, 1:2, and 1:4 (hN2:hN1) in the presence of either EDTA (10 mM) or ZnCl_2_ (50 μM). Analysis was performed at 20 °C and 50 000 rpm. The parameters obtained with SEDNTERP [26] were as follows: protein partial specific volumes (0.745, 0.733, and 0.739 ml/g for hN1, hN2, and hN1/2, respectively), buffer density (1.0059 and 1.006 g/ml for buffers containing EDTA and ZnCl_2_/CaCl_2_, respectively), and viscosity (1.0265 and 1.0228 mPa × s for EDTA and ZnCl_2_/CaCl_2_, respectively). Time-corrected data were analyzed with Sedfit software (version 16.1c) using the built-in continuous sedimentation coefficient distribution model, c(s). Maximum-entropy regularization of the c(s) model was set to a confidence level of 0.68 [27, 28].

### Isothermal titration calorimetry (ITC)

The binding of Zn(II) to hN1, hN2, and hN1/2 was monitored using a Nano-ITC calorimeter (TA Waters, USA) at 25 °C with a cell volume of 1 ml. All experiments were performed in buffer C. The hN1 and hN2 (titrands) concentrations were 0.1 mM, whereas the ZnSO_4_ (titrant) concentration was either 7 or 0.5 mM for the titration of hN1 and hN2 and hN1/2, respectively. The titrand and titrant concentrations were adjusted to obtain the best isotherms for proper analysis of equilibria. After temperature equilibration, successive injections of the titrant were made into the reaction cell in 5.22 μl increments at 300 s intervals with stirring at 200 rpm. Control experiments were performed to determine the heats of titrant dilution using identical injections of Zn(II) in the absence of protein.

The titration data were analyzed using NanoAnalyze (version 3.3.0), NITPIC (version 1.2.7) [29, 30] and SEDPHAT (version 15.2b) [31]. First, the data were preprocessed using NanoAnalyze software dedicated to the Nano-ITC calorimeter. Second, data integration and baseline subtraction were conducted using NITPIC freeware. Afterward, the integrated data were fitted with SEDPHAT. Titration of both proteins with Zn(II) was analyzed using the same binding model with one binding site: *A* + *B* ⇌ *AB*. The data were fitted first with constrained values obtained from prefitting in NanoAnalyze and subsequently revised in SEDPHAT with floating parameters of an incompetent fraction of protein and -log*K*_d_ with the corresponding Δ*H*_x_. The error estimates for the fitting results were produced using Monte Carlo analysis, individually for each experiment, with 500 iterations and a 0.9 level of confidence.

### Florescence spectroscopy

Stock solution of 8-Anilino-1-naphtalene-sulfonic acid (ANS) was prepared in dimethyl sulfoxide (DMSO) and the concentration of the fluorophore was verified at 376 nm with the extinction coefficient of 9140 1/(M × cm) [32]. Protein samples were prepared in buffer B supplemented with varying concentration of buffered ZnSO_4_ solution in a 384-well microplate (Greiner) and a total volume of 50 μl. Final concentration of nesfatins was 10 μM, and 50 μM of the fluorophore. All samples were prepared in triplicate. The microplate was incubated for 20 min at room temperature with shaking, centrifuged (1000 g, 2 min, 20 °C), and then scanned with BMG Clariostar Plus reader in a fluorescence intensity (FI) mode. ANS was excited at 350 nm and the fluorescence emission spectra were acquired from 375 to 650 nm. The data were averaged and smoothed with Savitzky-Golay filter for spectra presentation. Unsmoothed fluorescence intensity of the samples at 486 nm versus ZnSO_4_ concentration was fitted against Hill1 model with the Hill parameter left unrestricted.

### Zincon competitive titration

The binding of Zn(II) to hN1 and hN1/2 was further verified with Zincon (ZI), which forms a 1:1 complex with Zn(II) and has a published *K*_d_ value of 2.09 μM (pH 7.4) [33]. ZI stock solution was prepared by dissolving the chromophore in DMSO to a final concentration of 1 mM. Zincon affinity was verified under our experimental conditions by titrating 50 μM Zincon with ZnSO_4_. The absorbance at 618 nm was monitored with a Jasco V-630 spectrophotometer in a 1-cm quartz cuvette. The apparent chromophore *K*_d, ZI_ was calculated by fitting the data with OriginPro 2018 software against the quadratic equation. Competition experiments were performed by incubating various amounts of hN1 and hN1/2 suspended in buffer B with 50 μM ZI previously saturated with 50 μM ZnSO_4_. All samples were prepared in a 384-well microplate (Greiner) with a final volume of 100 μl. The microplate was incubated for 20 min at room temperature with shaking, centrifuged (1000 g, 2 min, 20 °C), and then scanned with a BMG Clariostar Plus plate reader in absorbance spectrum mode. The *K*_d_ of nesfatins was calculated as described by Kocyła et al. [33] using the *K*_d, ZI_ determined under our conditions.

## Results

### Intrinsic disorder prediction

The predictions for the IDRs of hN1, hN2, and hN1/2 was performed based upon their amino acid sequences. For this purpose, the PONDR VL-XT [18, 19] and DynaMine [20, 21] algorithms were implemented. The net mean charge of proteins plotted against hydropathy can be utilized to classify the proteins as ordered/disordered since both classes tend to create clusters that can be discriminated with a boundary, as first described by Uversky et al. [34]. hN1, hN2, and hN1/2 are situated very close to that boundary on the disordered side, as shown in Fig. 1B. This could indicate that the above proteins might contain both ordered and unordered regions. PONDR VL-XT was used to predict the IDRs in hN1/2 and in isolated hN1 and hN2. There are three segments that were predicted to be IDRs in hN1/2, which covered residues 1–6, 55–100, and 165–167. (Fig. 1C). In total, 32.9% of the hN1/2 structure was predicted to be disordered. There were also three regions predicted to be disordered in hN1, spanning 1–7, 55–75, and 84–85 aa. (Fig. 1E). This corresponds to 34.9% of the secondary structure content. Interestingly, after ‘excision’ of the hN2 peptide, the above results suggest that hN1 was structuralized between 75–84 aa. In the case of hN2, two disordered regions were identified that cover amino acids 1–16 and 81–83, which corresponds to 22.9% of the secondary structure content (Fig. 1E). These results indicate that each peptide might consist of alternating disordered/ordered regions and that the disorder propensity of the C-terminal region of hN1 is dependent on the presence of the hN2 fragment. Nonetheless, hN1 seems to have the most unordered structure in nature among the above peptides.

The DynaMine algorithm is a potent backbone dynamics predictor that calculates the S^2^ order parameter based upon a vast database of chemical shifts of proteins derived from NMR experiments. This parameter is sequence-dependent and is a measure of the N–H bond vector rotational freedom [20]. The output value can indicate a flexible region (disordered, S^2^ < 0.69), rigid (ordered, 0.8 < S^2^), or a region that can be viewed as an intermediate between the two (context dependent, 0.69 < S^2^ < 0.8) [20]. There are two short regions that were identified as disordered in each nesfatin (Fig. 1D, F) on the N-terminus and C-terminus and covered 1–7 aa (hN1, hN2, and hN1/2) and 81–86 (hN1), 77–83 (hN2), 161–167 aa (hN1/2). These short regions correspond well to those determined with the PONDR algorithms. However, the structural dynamics for each peptide between those regions are more convoluted from the DynaMine perspective, as the ordered and context-dependent regions are intertwined with each other. Taken together, these predictions point out the complex molecular properties of hN1, hN2, and hN1/2, which were further probed as described below.

### Secondary structure estimation and its changes under Zn(II) treatment

**Fig. 2:**
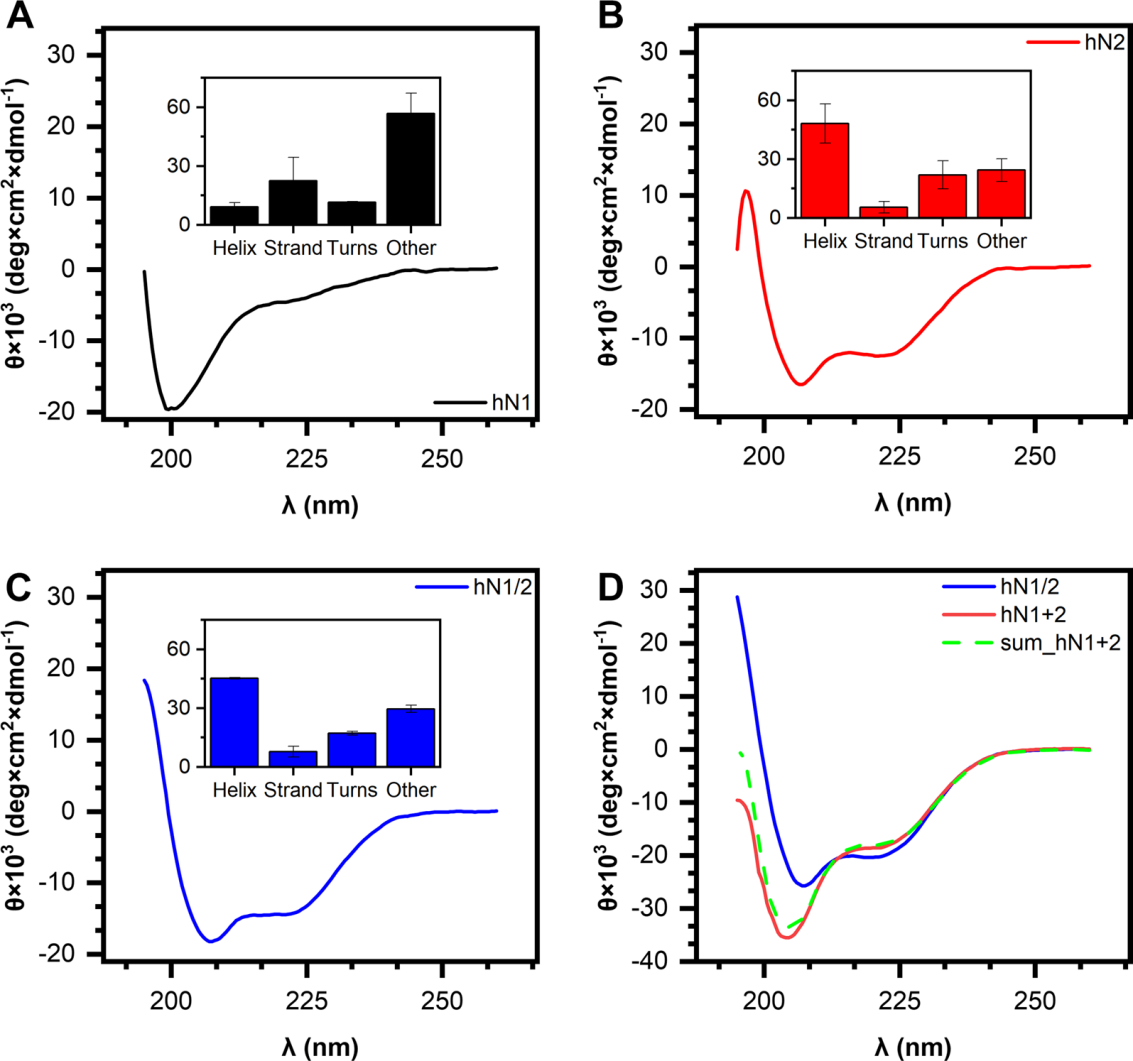
CD spectra of human nesfatins (0.15 mg/ml each). Averaged CD spectra of hN1 (**A**), hN2 (**B**), and hN1/2 (**C**). The inset graphs show secondary structure content estimated with CDPro software. (**D**) CD spectra of hN1/2 (solid blue line), a solution of mixed hN1 and hN2 (solid red line), and the algebraic sum of the individual spectra of the latter (dashed green line)

CD spectra in the far-UV region (195–265 nm) were recorded to validate the in silico prediction of the IDR content. The CD spectra of the apo-nesfatins are shown in Fig. 2. A strong negative maximum at 200 nm can be observed for the hN1 (Fig. 2A) spectrum, which is characteristic of highly disordered proteins [35]. On the other hand, the hN2 (Fig. 2B) and hN1/2 (Fig. 2C) spectra exhibit strong negative maxima at 208 and 222 nm, which is a characteristic feature of proteins with a high *α*-helical content [35]. This observation is supported by the results of the deconvolution of the spectra with the CDPro package (CONTINLL algorithm, Table 1). Unexpectedly, hN1 is even more disordered than suggested by in silico predictors, as 57 ± 10% of its secondary structure exists in this form. hN1 also contains a noticeable amount of *β*-strands (23 ± 12%). The structure of hN1 is thus very distinct from that of the remaining nesfatins, as hN2 and hN1/2 contain 48 ± 10% and 45.2 ± 0.4% *α*-helices, respectively. However, the contribution of IDRs is also significant and totals 24 ± 6% and 30 ± 2% for hN2 and hN1/2, respectively. The striking difference in the secondary structure content between free hN1 and hN2 along with the ordered nature of hN1/2 seems to indicate a strong effect of the hN2 fragment on the structure of hN1/2, since the N-terminal fragment covering hN1 is ordered due to the fragment.

**Table 1:** Estimation of the secondary structure content of human nesfatins. The data represent the mean ± standard deviation from 3 measurements

Protein	*α*-Helix (%)	*β*-Strand (%)	Turns (%)	Unordered (%)
hN1	9% ± 2	23% ± 12	11.4% ± 0.4	57% ± 10
hN2	48% ± 10	6% ± 3	22% ± 7	24% ± 6
hN1/2	45.2% ± 0.4	8% ± 3	17.2% ± 0.9	30% ± 2

To further examine this structural interdependency of both fragments, the spectrum for the mixed solutions of hN1 and hN2 (hN1 + 2) was acquired (Fig. 2D). The results show that this spectrum is complementary to the algebraic sum of the spectra of the free peptides. Surprisingly, the spectrum of hN1 + 2 is also distinct from the spectrum of hN1/2. This seems to be another indication of the observation that the molecular structure of hN1/2 is not a simple sum of the hN1 and hN2 structures. Thus, the above results augment the structural interdependency of hN1 and hN2. Moreover, hN2 may function as a structure-determining element when linked with hN1.

**Fig. 3:**
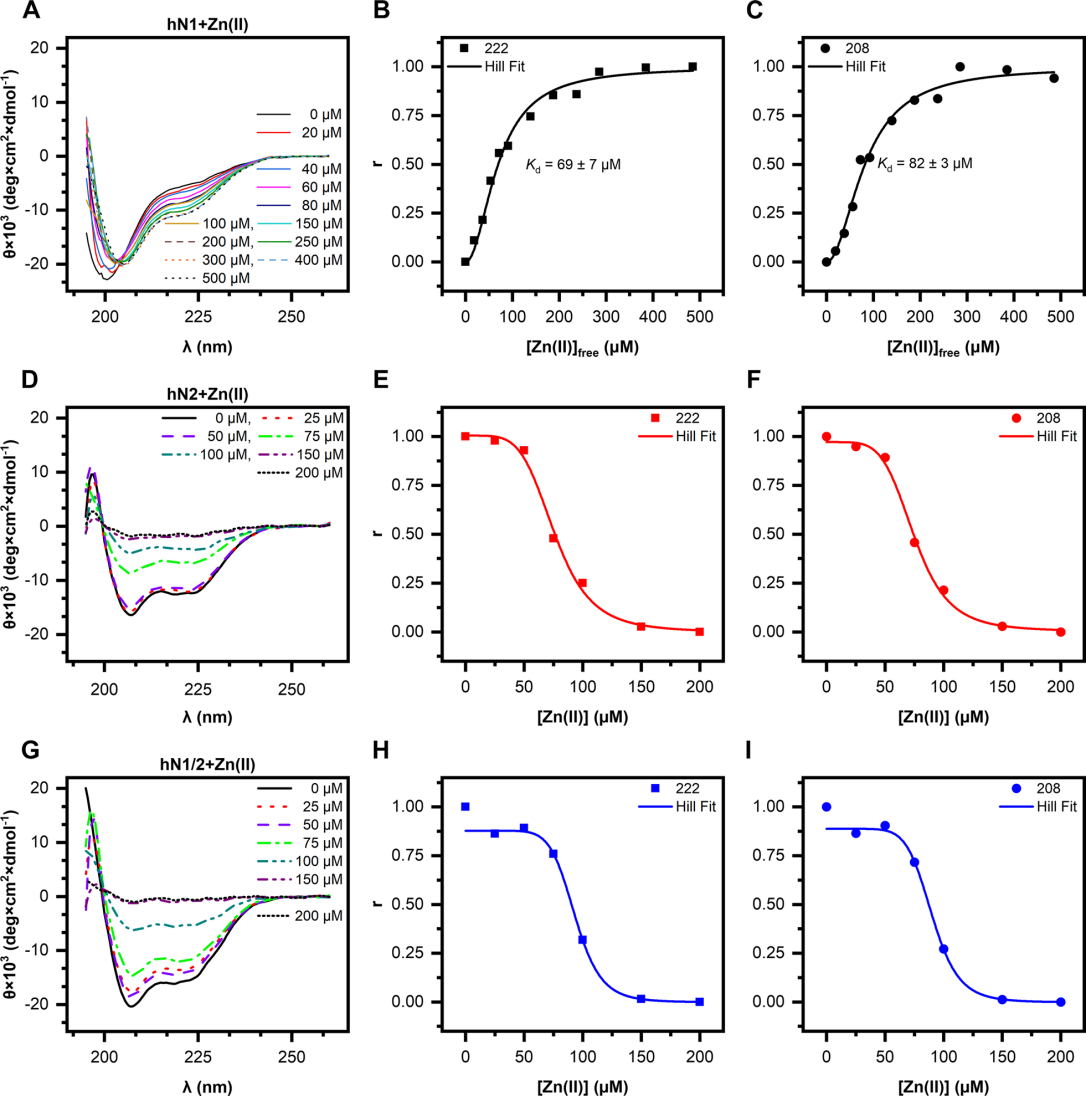
CD spectra of human nesfatins titrated with Zn(II) ions. (**A**, **D**, **G**) CD spectra of hN1, hN2, and hN1/2 acquired in the presence of varying concentrations of Zn(II) ions. (**B**, **C**) Fractional saturation of hN1 with Zn(II) ions and Hill fit of the data. Fractional saturation was calculated from the MRE at 222 (squares) and 208 nm (circles). (**E**, **F**) Relative abundance of hN2 in solution in the presence of Zn(II) ions and Hill fit of the data. (**H**, **I**) Relative abundance of hN1/2 in solution in the presence of Zn(II) ions and Hill fit of the data. Relative abundance was calculated from the MRE at 222 (squares) and 208 nm (circles) at different Zn(II) concentrations

Next, we tested the changes in secondary structure in the presence of Zn(II) and Ca(II), as their binding to the full-length protein has been reported [13, 17]. We observed no effect of Ca(II) on nesfatin spectra, as shown in Additional file 1: Fig. S3. However, the effect of Zn(II) was very profound, as shown in Fig. 3. The CD spectrum of hN1 titrated with Zn(II) (Fig. 3A) shows a strong redshift of the negative maximum at 200 nm to 208 nm and with simultaneous deepening of the maximum at 222 nm, both in a concentration-dependent manner. One isosbestic point at approximately 204 nm was also observed. The shift is associated with a disorder-to-order transition, as evidenced by the deconvolution of the spectra with the CDPro package (Table 2). There was a threefold increase in *α*-helix content during the titration of hN1, ranging from 10.0 to 31.2% with a simultaneous decrease in the disordered structure content from 72.5 to approximately 40.8%. With the titration curves fitted from the data acquired at 222 and 208 nm (Fig. 3B, C), the *K*_d_ values were estimated to be 69 ± 7 and 82 ± 3 μM, respectively. These values were further compared and correspond well with the results from ITC experiments (see below, “Isothermal titration calorimetry" section).

**Table 2:** Secondary structure changes of human nesfatin-1 in the presence of Zn(II) ions

ZnCl_2_ (μM)	*α*-Helix (%)	*β*-Strand (%)	Turns (%)	Other (%)
0	10.0	10.3	7.2	72.5
20	17.2	24.9	16.4	41.5
40	18.6	13.5	13.4	54.5
60	22.4	21.3	18.2	38.1
80	24.7	16.4	16.5	42.4
100	22.9	7.0	14.4	55.7
150	28.0	11.2	16.5	44.3
200	32.1	13.3	16.4	38.2
250	32.2	11.5	17.4	38.9
300	32.3	10.0	17.0	40.7
400	35.0	12.4	18.3	34.3
500	31.2	9.4	18.6	40.8

The behavior of hN2 (Fig. 3D) and hN1/2 (Fig. 3G) titrated with Zn(II) is different from that of hN1. The presence of Zn(II) strongly destabilizes both peptides, as evidenced by the concentration-dependent loss in CD signal, which was presumably due to the aggregation and/or precipitation of the peptides. The curves fitted to data at 208 and 222 nm versus Zn(II) concentration for hN2 (Fig. 3E, F) and hN1/2 (Fig. 3H, I) are in fact similar to those typically observed for competition binding experiments [36]. Moreover, the obtained curves indicate evident cooperativity of Zn(II) binding, which in turn leads to precipitation and/or aggregation of hN2 and hN1/2. With the fitted curves, the Zn(II) concentrations were also estimated to have a 50% CD signal loss of 75 ± 3 and 90 ± 4 μM for hN2 and hN1/2, respectively. Hence, to prevent hN2 and hN1/2 precipitation in the AUC experiments (see below), it was critical to choose a Zn(II) concentration of 50 μM corresponding to a CD signal above 80%.

### Quaternary structure changes of nesfatins in the presence of Zn(II) ions measured with SV-AUC

The presence of Zn(II) induced significant changes in the secondary structure of human nesfatins, and the presence of Ca(II) did not induce significant changes, as discussed in the previous paragraph. Hence, to evaluate whether these observations are accompanied by tertiary and/or eventually quaternary structure changes, we performed SV-AUC experiments.

**Fig. 4:**
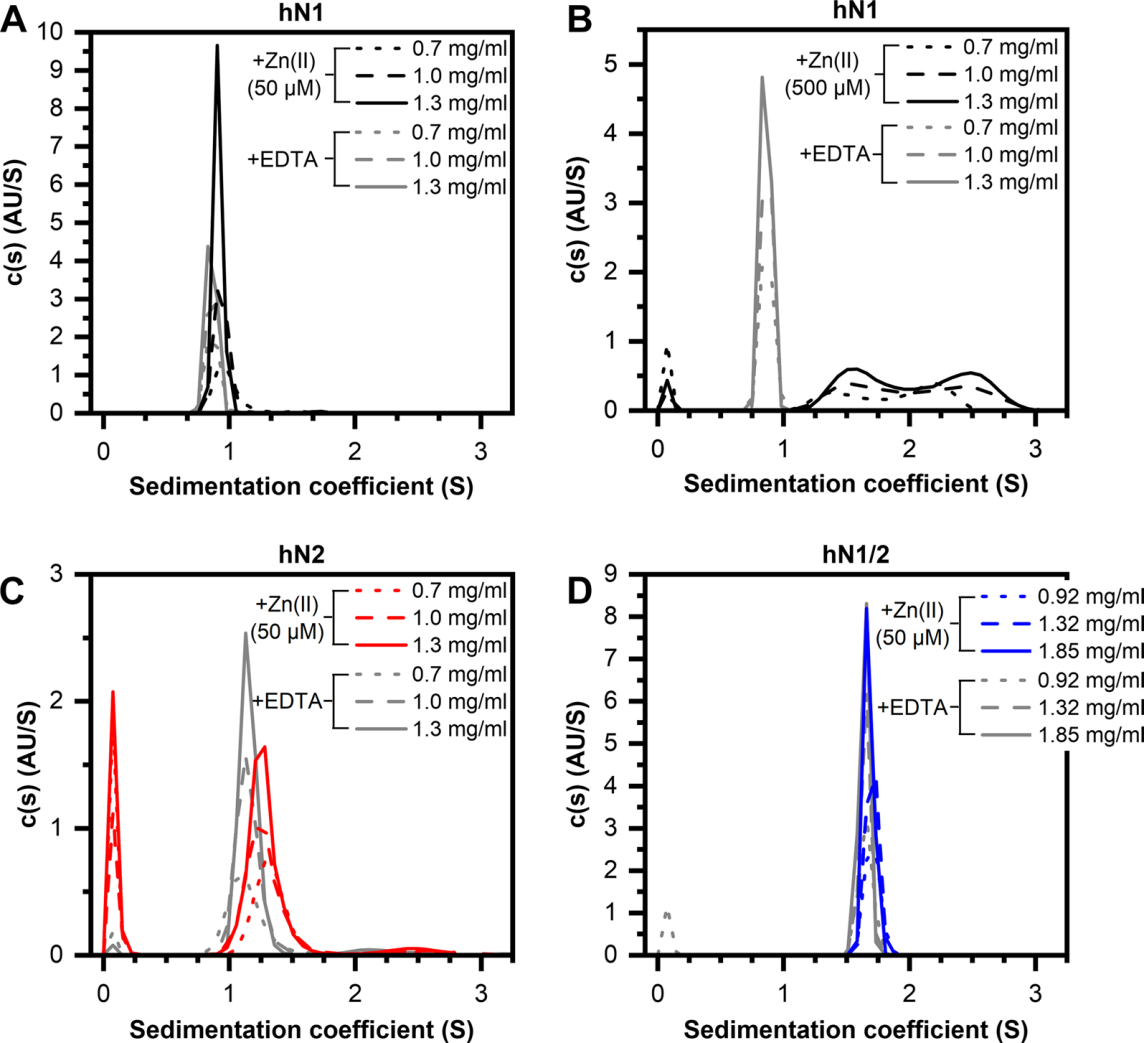
SV-AUC analysis of human nesfatins in the presence of 50/500 μM Zn(II) and 5 mM EDTA (gray line). (**A**, **B**) Distribution plot of hN1 (black line), hN2 (**C**, red line), and hN1/2 (**D**, blue line). The data were recorded at 1.3 mg/ml (solid line), 1.0 mg/ml (dashed line) and 0.7 mg/ml concentrations (dotted line) of hN1 and hN2 and at 1.85 mg/ml (solid line), 1.32 mg/ml (dashed line), and 0.92 mg/ml (dotted line) of hN1/2

The SV-AUC data (Fig. 4) revealed that in the absence of Zn(II) (5 mM EDTA), hN1 (Fig. 4A, B), hN2 (Fig. 4C), and hN1/2 (Fig. 4D) display continuous sedimentation coefficient distributions c(s) that were very well-defined and spike-like. The s_(20,w)_ values for these proteins oscillate approximately 0.91S for hN1, 1.2S for hN2, and 1.73S for hN1/2 in each cell (Table 3). The calculated apparent molecular mass (M_app_) was approximately 10.9 kDa for hN1 and hN2 and 21 kDa for hN1/2, which is in very good agreement with the results obtained from the MS experiments (Additional file 1: Fig. S2). All proteins under these conditions sediment primarily in a monomer form; however, there is a small fraction of higher oligomers in the cells containing hN2 with an M_app_ of approximately 33 kDa, which could indicate some aggregation or the presence of a trimer. The obtained f/f_0_ coefficient values were found to be 1.8 for hN1 and 1.5 for both hN2 and hN1/2. The f/f_0_ value indicates the sphericity of the molecules, as for globular proteins, which is typically found in the 1.2–1.4 range, whereas higher values indicate more open extended geometries [37]. Thus, the f/f_0_ coefficient values allow us to presume that hN1 has a long ellipsoidal shape. This result seems to be another confirmation of the disordered nature of hN1, as revealed in “In silico molecular analysis” section, because IDPs display unusually high f/f_0_ values due to their large size-to-mass ratio [37]. In contrast, hN2 and hN1/2 are more akin to globular proteins, based on the values of the f/f_0_ parameters. These results show that hN1 in the context of hN2 (hN1/2) displays a more globular shape. Conversely, free hN1 displays an extended and cylindrical shape. Taken together, the results indicate that that proteolytic processing of hN1/2 may result in the formation of two structurally different products, in which the molecular properties are very distinct when compared to those of the conjoined hN1 and hN2.

**Table 3:** Hydrodynamic properties of human nesfatins

Protein	Compound	c [mg/ml]	rmsd	s_(20, w)_ [S]	f/f_0_	R_h_ [nm]	M_app_ [kDa] (%)
hN1	(5 mM) EDTA	0.7	0.006130	0.91	1.82	2.69	10.8 (100)
		1.0	0.006342	0.91	1.84	2.72	11.0 (100)
		1.3	0.006572	0.90	1.84	2.72	10.9 (100)
	(500 μM) ZnCl_2_	0.7	0.008389	1.53 2.22	1.23	1.93 2.33	13.0 (42) 23.0 (58)
		1.0	0.009239	1.73 2.53	1.22	2.02 2.45	15.5 (55) 27.5 (45)
		1.3	0.010125	1.70 2.51	1.24	1.99 2.42	14.5 (49) 26.0 (51)
hN2	(5 mM) EDTA	0.7	0.005973	1.19 2.52	1.54	2.31 3.37	11.7 (92) 36 (8)
		1.0	0.006112	1.20 2.56	1.46	2.14 3.13	10.9 (93) 34.1 (7)
		1.3	0.006329	1.20 2.30	1.44	2.10 2.90	10.8 (93) 28.3 (5)
	(50 μM) ZnCl_2_	0.7	0.006531	1.38 2.72	1.42	2.20 3.09	12.9 (92) 35.6 (8)
		1.0	0.006628	1.33 2.74	1.43	2.19 3.14	12.3 (92) 36.5 (8)
		1.3	0.007021	1.32 2.59	1.42	2.16 3.02	12.1 (93) 33.1 (7)
hN1/2	(5 mM) EDTA	0.92	0.006636	1.73	1.52	2.78	21 (100)
		1.32	0.007039	1.73	1.53	2.80	21 (100)
		1.85	0.007511	1.72	1.54	2.82	21 (100)
	(50 μM) ZnCl_2_	0.92	0.006471	1.78	1.49	2.74	21 (100)
		1.32	0.007108	1.78	1.50	2.77	21 (100)
		1.85	0.007529	1.75	1.52	2.79	21 (100)

The numbers in the round bracket represent the percentage of each fraction relative to the two main sedimenting species (100%)

In the presence of Ca(II), we did not observe any significant changes in the c(s) distribution (Additional file 1: Fig. S4). However, the addition of 50 μM Zn(II) has a very subtle effect on the c(s) distribution. There was a slight shift in the s_(20,w)_ parameter for hN1 (Fig. 4A), hN2 (Fig. 4C), and no shift occurred for hN1/2 (Fig. 4D). It is therefore worth noting that the c(s) distribution of hN2 is Zn(II)-sensitive, whereas the distribution of hN1/2 appears to be insensitive to Zn(II). This observation highlights the greater susceptibility of free hN2 to Zn(II) compared to that of hN1/2. Overall, it appears that these conditions do not have a strong influence on the tertiary/quaternary structure, based on the similarity of the obtained parameters with those from samples supplemented with EDTA. However, the c(s) distribution of the hN1 sample supplemented with 300 and 500 μM Zn(II) (Additional file 1: Fig. S5 and Fig. 4B, respectively) is dramatically distinct. Two populations of sedimenting species emerged in a Zn(II) concentration-dependent manner with a substantial shift in the s_(20,w)_ parameter from 0.9S to 1.4/1.53 and 2.1/2.4S (Additional file 1: Table S1, Table 3 for 300/500 μM Zn(II), respectively). The s_(20,w)_ parameter change is accompanied by an increase in the M_app_ from 10.9 kDa to approximately 14.3 and 25.5 kDa, indicative of the formation of a dimer by holo-hN1. There is also a significant reduction in the f/f_0_ parameter from 1.8 to 1.23 for Zn(II)-saturated hN1, which is strongly evident of structural compaction; this is possibly due to the adoption of a more globular shape, which is in good agreement with the results of CD spectroscopy (see “Secondary structure estimation and its changes under Zn(II) treatment” section). We also probed for hN1/hN2 interactions with SV-AUC experiments (Additional file 1: Fig. S6). However, there was no evidence of tertiary and quaternary structure changes that could be the result of hN1/hN2 interactions. The observed c(s) sedimentation distribution in the presence/absence of Zn(II) was consistent with the distributions observed for the isolated hN1 and hN2, as described earlier. Thus, we concluded that based on CD (“Secondary structure estimation and its changes under Zn(II) treatment” section) and SV-AUC experiments, hN1 and hN2 did not interact under our conditions in vitro.

These results show that the apo-hN1 exists primarily in a monomer form, while the holo-hN1 exhibits a propensity for dimerization due to the Zn(II) concentration-dependent disorder-to-order transition. It is also worth noting that similar effects of compaction and oligomerization under Zn(II) and Ca(II) treatment were reported by Skorupska et al. for hNucb2 and ggNucb2 [13, 17].

### Isothermal titration calorimetry

To accurately determine the thermodynamic parameters of Zn(II) binding by hN1, hN2, and hN1/2 and to compare them, we performed isothermal titration calorimetry experiments.

**Fig. 5:**
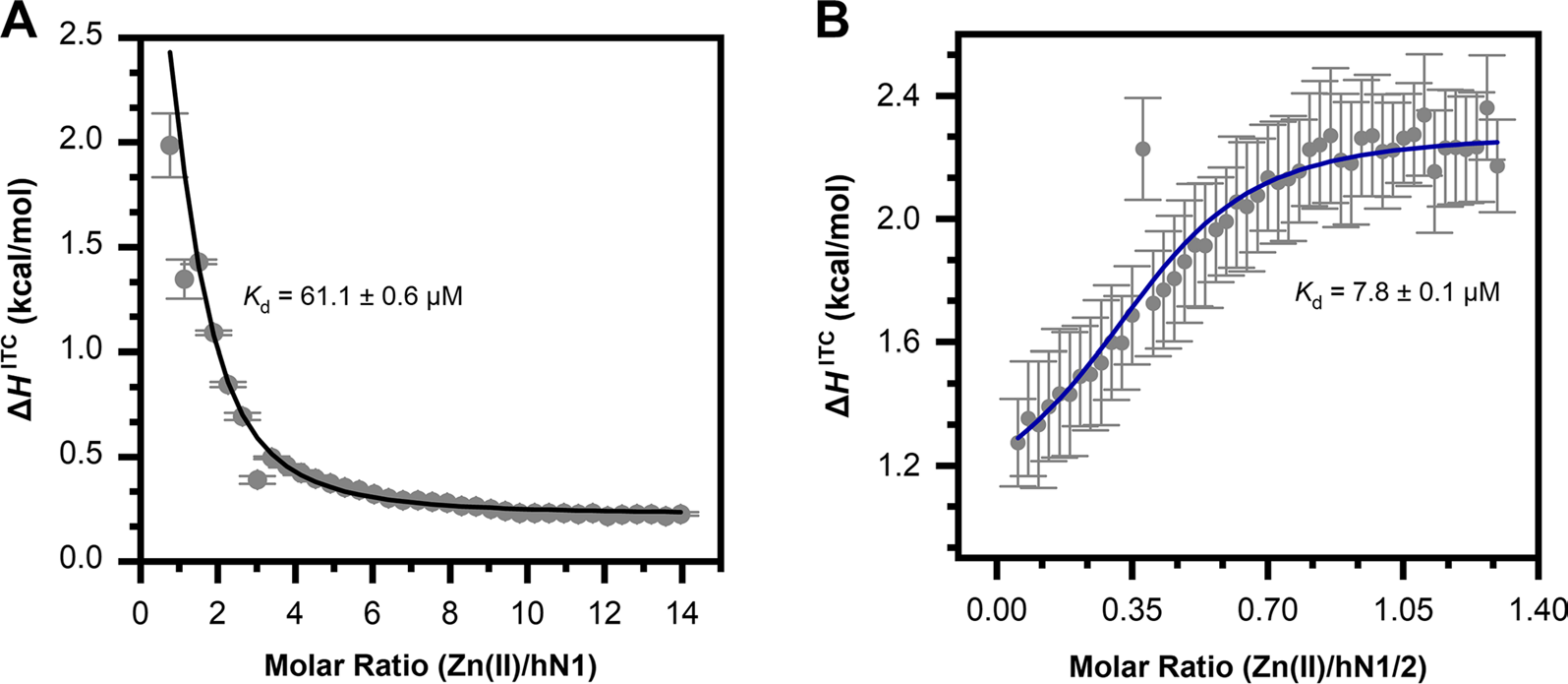
ITC results of human nesfatin-1 and -1/2 titrated with Zn(II) in 20 mM MES, pH 6.5; 150 mM NaCl, represented as functions of measured enthalpy changes Δ*H*.^ITC^ plotted against molar ratios of titrant to titrand. (**A**) Binding isotherm of 0.1 mM hN1 titrated with 7 mM Zn(II). (**B**) Binding isotherm of 0.1 mM hN1/2 titrated with 0.5 mM Zn(II)

The binding of Zn(II) to hN1 and hN1/2 resulted in very different thermograms. Titration of hN1 with Zn(II) is characterized by prominent endothermic heats that plateau at a very high molar ratio of Zn(II)/hN1 (Additional file 1: Fig. S7A). Such behavior suggests a low affinity of the titrate, which was confirmed by the fitted *K*_d_ value of approximately 61 μM (Fig. 5A). The endothermic binding process, which is represented by the fitted Δ*H*^ITC^ value of approximately 5 kcal/mol, is, however, complemented by the substantial positive entropic factor, thus resulting in an overall negative Gibbs enthalpy of approximately -5.75 kcal/mol (Table 4). The titration reaction was fitted to the one-binding site model in which the incompetent protein fraction remained at 0, which suggests that hN1 interacts with Zn(II) to form a complex with a 1:1 stoichiometry per monomer of the protein.

**Table 4:** Thermodynamic parameters of Zn(II) complexation with hN1 and hN1/2 derived from fitting data to the AB model of interactions

Parameter	hN1 + Zn(II)	hN1/2 + Zn(II)
[hN1]/[hN1/2] (µM)	100	100
[Ζn(II)] (µM)	7000	500
Χ^2^	1.04	1.22
*K*_d_ (µM)	61.1 ± 0.6	7.8 ± 0.1
Δ*H* (kcal/mol)	5.2 ± 0.4	− 1.2 ± 0.2
TΔ*S* (kcal/mol)	10.908	5.725
Δ*G* (kcal/mol)	− 5.748	− 6.965
Inc. protein fraction	0	0.57 ± 0.08

The hN1/2 Zn(II) binding process varies substantially compared to that of its shorter predecessor. First, the metal ion binding thermogram shows negative heats (of the binding reaction with respect to the equilibrium heats, Additional file 1: Fig. S7B), suggesting that the complexation reaction for hN1/2 is an exothermic process. Moreover, hN1/2 interacts with Zn(II) more avidly, which is represented by much faster saturation on the Zn(II)/hN1/2 molar ratio scale (Fig. 5B). In fact, fitting to the same model of interaction resulted in a *K*_d_ value of approximately 7.8 μM, which is almost 10 times lower than the dissociation constant for holo-hN1. Overall, the Gibbs enthalpy of Zn(II) complexation by hN1/2 is more favorable compared to that of hN1 and is approximately − 7 kcal/mol, which exhibits a much more dominant enthalpic contribution compared with that of the hN1 reaction (Δ*H*^ITC^ ≈ − 1.2 kcal/mol). The final difference between these two proteins pertains to complex stoichiometry, which in the case of hN1/2 with Zn(II) was fitted to be 2:1 (Zn(hN1/2)_2_), based on the incompetent protein fraction value of approximately 0.5.

Unfortunately, due to the high destabilization of hN2 under the ITC conditions, we could not use the obtained data. However, we did observe endothermic peaks that were indicative of probable interactions, which were further verified with ANS binding experiments (see “ANS binding to nesfatins in the presence of Zn(II) ions” section).

### ANS binding to nesfatins in the presence of Zn(II) ions

Previous experiments proved that hN1 undergoes major structural rearrangements under Zn(II) treatment (see “Secondary structure estimation and its changes under Zn(II) treatment” and “Quaternary structure changes of nesfatins in the presence of Zn(II) ions measured with SV-AUC” sections). In contrast, in the presence of Zn(II), hN2 and hN1/2 exhibited a tendency to precipitate (see “Secondary structure estimation and its changes under Zn(II) treatment” section), but unexpectedly, the ITC results showed that hN1/2 does bind Zn(II) (see “Isothermal titration calorimetry” section). To further probe Zn(II)-induced structural changes in nesfatins, we performed 8-aniline-1-napthalene-sulfonic acid (ANS) fluorescence spectroscopy experiments. ANS is a fluorophore that is characterized by a low quantum yield in an aqueous environment. However, upon binding to exposed hydrophobic sites of the proteins, ANS exhibits increased fluorescence intensity and an observable blueshift in the spectrum [38]. In this context, we examined whether Zn(II) ions also induce exposure of the hydrophobic core of nesfatins.

**Fig. 6:**
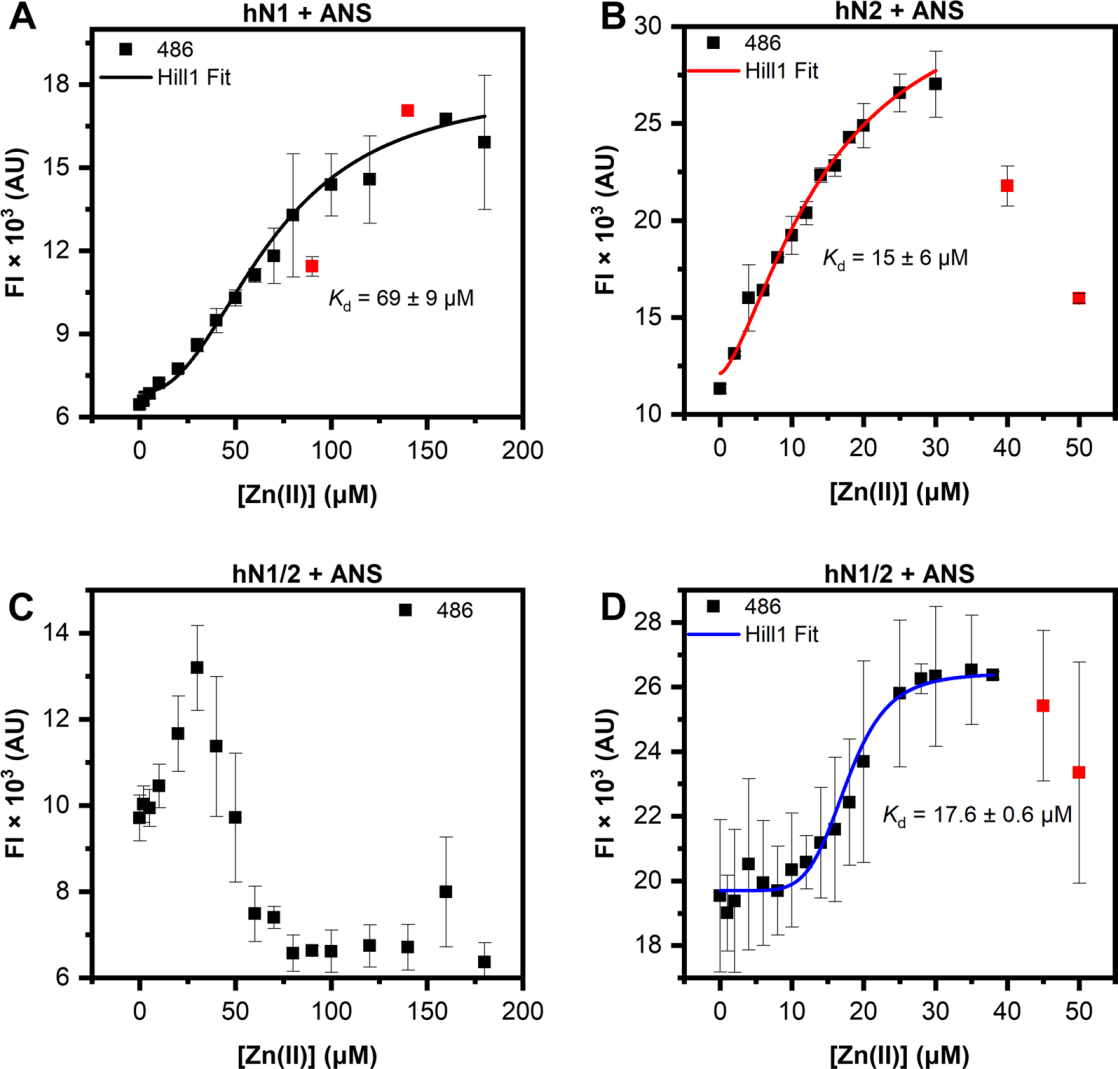
Changes in ANS emission intensity at 486 nm upon titration of (**A**) hN1, (**B**) hN2, and (**C, D**) hN1/2 with Zn(II) ions. The red squares represent data points excluded from fitting

As expected, free ANS excited at 350 nm had the lowest observed fluorescence intensity (FI), with a maximum at 517 nm (Additional file 1: Fig. S8). In contrast, the ANS emission spectra in the presence of hN1 titrated with Zn(II) ions (Additional file 1: Fig. S8A) showed a concentration-dependent hypsochromic shift of the spectrum, which was accompanied by an increase in the FI. During the titration, the FI was initially at the level of the free ANS, which indicates that apo-hN1 does not possess exposed hydrophobic surfaces. The hydrophobic sites were uncovered gradually with the addition of Zn(II) ions. The apparent *K*_d_ was estimated to be 69 ± 9 μM by utilizing the fitting of the FI data at 486 nm versus Zn(II) concentration (Fig. 6A), and this value corresponds very well to the results from CD spectroscopy (“Secondary structure estimation and its changes under Zn(II) treatment” section) and ITC experiments (“Isothermal titration calorimetry” section). The emission spectra of ANS in the presence of hN2 and Zn(II) ions (Additional file 1: Fig. S8B) were also blueshifted with a concentration-dependent intensity surge. It is also worth noting that the initial FI was higher than that of samples containing hN1, and the intensity spike was more robust. These results point to a broader availability of the hydrophobic residues of apo-hN2 as well as their more eager exposure under Zn(II) ion treatment. Moreover, after the Zn(II) concentration was above 30 μM, an intensity drop was also observed in CD experiments (“ Secondary structure estimation and its changes under Zn(II) treatment” section). Fitting the data at 486 nm (Fig. 6B) revealed that Zn(II) is possible bound by hN2 and the apparent *K*_d_ was estimated to be 15 ± 6 μM. Similar results were obtained for hN1/2; the emission spectra (Additional file 1: Fig. S8C, S8D) were also blueshifted to 486 nm, which was complemented by the Zn(II) concentration-dependent spike in the FI. Additionally, hydrophobic residues in hN1/2 seem to be the most exposed among the studied nesfatins, based on the highest FI difference between the apo-form and free ANS. On the other hand, after the Zn(II) concentration was greater than 40 μM, an FI drop is observed again, which corresponds well to the results from CD experiments (“Secondary structure estimation and its changes under Zn(II) treatment” section). Since the obtained FI data at 486 nm (Fig. 6C) exhibited biphasic character, we again probed the 0–50 μM range, which was indicative of the probable binding of Zn(II) ions. The curve fitted to that data (Fig. 6D) was strongly cooperative, and the calculated apparent *K*_d_ was 17.6 ± 0.6 μM, which corresponds to the value obtained from the ITC experiment (“Isothermal titration calorimetry” section). Taken together, these results augment the effects of Zn(II) on the structure of nesfatins, their ability to bind them and especially the Zn(II) sensitivity of hN2, which seems to translate to hN1/2.

### Zincon competitive titration

**Fig. 7:**
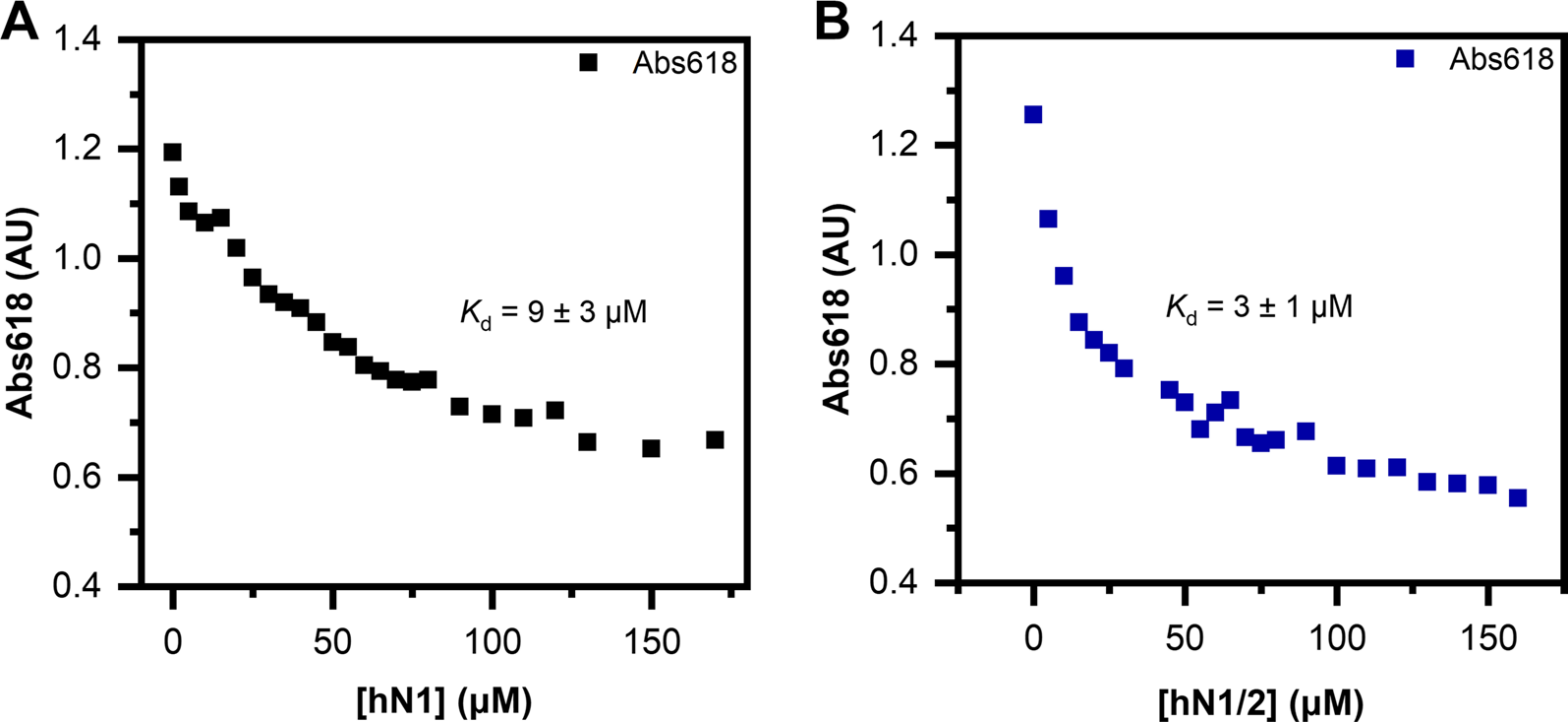
Competitive titration of Zincon (50 μM) saturated with 50 μM ZnSO_4_ with human nesfatins. (**A**) Titration of Zincon with hN1. (**B**) Titration of Zincon with hN1/2

To further confirm the ability of hN1 and hN1/2 to bind Zn(II), we performed competitive titration experiments using Zincon (50 μM) saturated with Zn(II) (50 μM). For this purpose, we determined the apparent dissociation constant of the chromophore under our buffer conditions to obtain the value of *K*_d,ZI_ = 1.3 ± 0.2 μM, which is in good agreement with the 2.09 μM reported in the literature [33]. We observed a concentration-dependent absorbance reduction at 618 nm under hN1 treatment (Fig. 7A), which further demonstrates its ion binding capability. The apparent *K*_d_ was calculated according to Kocyła et al. [33] and was found to be 9 ± 3 μM, which deviates from the previous results, possibly due to the more complicated stoichiometry of the actual model [33]. As expected, the competitive effect of hN1/2 on the Zn(II)-ZI complex is stronger than that of hN1 due to its higher affinity for Zn(II). The calculated *K*_d_ was 3 ± 1 μM, which corresponds to the values obtained from previous studies. In conclusion, we once more demonstrated the Zn(II) binding properties of hN1 and hN1/2, which also underlines their potential involvement in Zn(II) homeostasis.

## Discussion

Herein, we present new insight into the structural characterization and interactions of the products from the proteolytical processing of hNucb2, namely, hN1, hN2, and hN1/2. To the best of our knowledge, this is also the first description of the molecular properties of these peptides. In the first stage of our studies, we performed in silico analysis of human nesfatins, which revealed their possible mosaic-like character with intertwined disordered-ordered regions; the results were similar to the results described by Skorupska et al. for hNucb2 [13]. Moreover, the results suggested that among the products, hN1 is the most disordered in nature, raising initial considerations of structural interdependency between hN1 and hN2.

For this reason, CD spectroscopy was employed to gain insight into secondary structure content, and the obtained data agreed with the in silico results. The content of IDRs in hN1 was 57 ± 10%, and *β*-strands were also found to have a strong contribution (23 ± 12%) to the secondary structure of hN1. Therefore, these results prove that hN1 is indeed a member of the IDPs family, and it might perform the diverse physiological effects through similar mechanisms discussed earlier. Moreover, ubiquitous central and peripheral expression of Nucb2/N1 throughout the body further underlines its biomedical importance. The peptide was found in the central nervous system in the hypothalamic nuclei as follows: arcuate, paraventricular, supraoptic, and dorsomedial nucleus, as well as in other brain centers such as the brainstem [12, 39]. Expression of Nucb2/N1 extends further to the gastrointestinal tract, i.e., gastric mucosal glands, a submucosal layer of the duodenum [40, 41], and B cells in the pancreatic islets [42]. Other tissues that display Nucb2/N1 immunoreactivity include adipocytes [43], testis [44], and heart [45]. The broad distribution of this peptide in concert with its IDP properties described here seems to be partly responsible for its auto, paracrine, and tissue-specific mode of action.

The secondary structure of the remaining peptides was very distinct compared to that of hN1. CD experiments revealed that hN2 and hN1/2 exhibit ordered characteristics with *α*-helical contents of 48 ± 10% and 45.2 ± 0.4%, respectively. The content of IDRs was also noticeable and totaled 24 ± 6% and 30 ± 2% for hN2 and hN1/2, respectively. These results were complementary to the in silico analysis. Moreover, the results raise the question of whether in vivo proteolytical processing of hN1/2 could function as an activation mechanism that enables hN1 interaction with multiple targets through the transition from a mostly ordered to an unordered structure. For this reason, we decided to probe for mutual hN1 and hN2 interactions in solution with CD and SV-AUC experiments. CD spectroscopy revealed no changes in the secondary structure that could result from the interactions between hN1 and hN2, based on the difference between the spectrum of hN1/2 and the overlapping spectra of the mixed solution of hN1 + 2 and their algebraic sum. Thus, free hN1 indeed exhibits different molecular properties than those when linked covalently with hN2, and the mere presence of free hN2 in solution is insufficient to induce any structural changes. In addition, we also did not observe significant changes in the c(s) distribution of the solution of hN1 + 2 at any w/w ratio in SV-AUC experiments, both in the presence and absence of Zn(II) ions. Therefore, based on the lack of changes in the secondary, tertiary, and quaternary structures, we conclude that there is no interaction between free hN1 and hN2 in vitro under our conditions. This observation further illustrates the interdependency between the hN1 structure (and possibly function) and hN2 that translates to the properties of hN1/2 and hNucb2. Hence, hN2 could function as a molecular switch that turns the appropriate molecular character of hN1 on and off.

Metal ions represent an important group of signal transducers and effectors and are essential cofactors of many proteins. Among them, one of the most biologically important metal ions is Zn(II). This trace element is the second most abundant d-block metal in living organisms after iron [46]. Zn(II) plays a major role in the regulation of many cellular processes, such as proliferation [47], apoptosis [48], regulation of immune responses [49, 50], the pathogenesis of cardiovascular [51] and neurodegenerative diseases [52], and carcinogenesis [53]. Proteins that contain Zn(II) are abundant in the human organism. In fact, 3% of the human genome encodes zinc finger (ZF) proteins, which is an impressive pool of the human genome [54]. Moreover, bioinformatic analysis showed that 10% of the human proteome might consist of Zn(II)-binding proteins [55]. However, the above bioinformatic studies did not consider transient Zn(II)-dependent protein–protein interactions, which have recently been recognized as important cell modulators [56]. ZF proteins are structurally organized under the influence of Zn(II) and unfold upon Zn(II) dissociation [57]. However, the classification of Zn(II)-binding proteins is quite complicated [58]. Additionally, a number of other proteins are continually being reported to naturally bind Zn(II) and exhibit activity and/or stability due to some structural motifs, which cannot necessarily be identified as characteristic.

Since the binding of Zn(II) by the N-terminal fragment of hNucb2 [17] and Ca(II) by the C-terminal fragment [13] was reported, we decided to investigate the effects of those ions on the structure of nesfatins. In the first stage, we assessed the changes in the secondary structure in the presence of the above ions with CD spectroscopy. As expected, Ca(II) did not have any influence on the secondary structure of nesfatins, nor did we observe changes in the tertiary/quaternary structure in SV-AUC experiments. These results are consistent with those published by Skorupska et al. [13]. On the other hand, we did observe the strong influence of Zn(II) on the structure and stability of nesfatins throughout all experiments, which also proved the ability of nesfatins to recognize and bind Zn(II). Deconvolution of the CD spectra of hN1 titrated with Zn(II) showed a strong concentration-dependent increase in the *α*-helical content. Moreover, with the fitting of the data at 208 and 222 nm, the apparent *K*_d_ was estimated, which was in good agreement with the ITC and fluorescence spectroscopy results. The ITC results also showed a 1:1 binding model for hN1-Zn(II), corresponding to one bound ion per monomer. We also demonstrated that Zn(II) binding is associated with a disorder-to-order transition, a common feature of IDPs. These structural changes were also observed in SV-AUC experiments. The value of the f/f_0_ parameter for apo-hN1 indicates its elongated form, while holo-hN1, in the presence of Zn(II), undergoes compaction due to the adoption of a more globular structure. The SV-AUC results also showed that Zn(II)-directed dimerization of hN1 occurs, which in turn could be a prerequisite for interactions with specific ligands in vivo. Surprisingly, the ITC results revealed a 2:1 binding stoichiometry (hN1/2:Zn(II)) of hN1/2, but we did not observe oligomerization of this protein under SV-AUC conditions. This could be the result of the different buffer conditions and/or precipitation during the ITC experiment.

The above observations were complemented by the Zn(II)-induced hydrophobic surface exposure observed in the presence of ANS. It is also worth noting that hN1, in the absence of Zn(II), displayed the lowest ANS binding compared to that of the remaining peptides, resulting from its unordered structure, which lacks defined hydrophobic pockets that are suitable for fluorophore binding. However, structurization under Zn(II) treatment leads to the gradual formation of hydrophobic sites, resulting in increased ANS fluorescence. Zn(II) had a dual influence on the hN2 and hN1/2 structures. We first observed gradual exposure of the hydrophobic sites for both peptides followed by a decrease in fluorescence intensity, which resulted from the precipitation that was also observed after exceeding the Zn(II) threshold concentration in the CD studies. This experiment allowed for the apparent *K*_d_ to be estimated. The obtained value for hN1/2 was in good agreement with the ITC results. Moreover, since we could not obtain the apparent *K*_d_ of hN2 from the ITC data, this experiment proved our assumptions that were derived from the former experiment, i.e., that hN2 can bind Zn(II). It is also worth noting that the highly cooperative character of the binding curve of hN1/2 might result from the antagonistic mechanisms of hN1 and hN2 for Zn(II) binding and precipitation, respectively. This raises the question of whether Zn(II)-driven destabilization of hN1/2 and full-length hNucb2 [17] might be the effect of hN2-directed aggregation. Moreover, in silico studies of Nucb2 revealed that the aggregation prone region is located in the N-terminal segment, which was proven in this paper [17]. ZI competitive titration also proved the ability of hN1 to bind Zn(II), although the obtained *K*_d_ was lower than expected, possibly due to a more complicated stoichiometry in the actual model. However, the data obtained for hN1/2 correspond well to the previous experiments.

Nucleobindins are a highly conserved protein family that is also expressed in non-mammals, e.g. goldfish (*Carassius auratus*) [59, 60], zebrafish (*Danio rerio*) [61], frog (*Microhyla ornata*) [62], in hypothalamus, pituitary, other brain centers, and peripheral tissues. Broad distribution along with multidomain structure of Nucleobindin-1 (Nucb1; Nucb2 paralog)/Nucb2 is reflected by their multifunctionality. Both orthologs are engaged in Ca(II) homeostasis [63, 64], G protein signaling [65], and nesfatin-1 like peptide (NLP; a product of proteolytical processing of Nucb1) was shown to be involved in energy expenditure in goldfish [60]. The results presented here suggest that human nesfatins might function as Zn(II) sensors. This function might be universal, given highly conserved amino acid sequence of Nucb1/Nucb2 and products of their proteolytical processing (NLP and nesfatins, respectively). Hence, Zn(II)-binding might modulate Nucb1/Nucb2, NLP/nesfatins activity in other vertebrates in a similar manner as described here. However, the purpose of Zn(II)-sensing by nesfatins remains unclear, especially in the context of the values for the determined *K*_d_ and in relation to Zn(II) cellular levels. It appears that proteolytical processing does not substantially influence the *K*_d_ value, which is still in the 10^–5^-10^–6^ M range, as reported for hNucb2 [17]. Zn(II) has a very strong influence on the structure and stability of nesfatins, which in turn might regulate their bioactivity. Elevation of Zn(II) levels in the cytoplasm is counteracted by the influx of these ions into the endoplasmic reticulum and Golgi apparatus (GA) [66]. Interestingly, the Leu/Ile rich region of hNucb2 and human Nucb1 (hNucb1) is considered to be a novel Golgi retention motif and suggests localization of hNucb2/nesfatin-1/2 in this organelle [67]. Indeed, it was shown that Nucb1 lacking the N-terminal sequence was residing in the cytosol [68].

Above findings, along with the distinct mechanism of Zn(II) binding by hN1/2 (proprotein) presented here, indicates that the physiological effects of Nucb2/N1/2 and Nucb1/NLP might also be evoked by their subcellular localization. Moreover, since PC1/3 undergoes maturation in GA [69], its proteolytical disruption of the Leu/Ile motif of Nucb2/N1/2 and Nucb1 in this organelle might initiate N1, N2, and NLP subcellular trafficking and/or their release via the secretory pathway. Hence, this mechanism might be responsible for inducing both auto and paracrine effects observed in different species for the orthologs.

The brain regions that constitute the limbic system include the amygdala, hypothalamus, limbic cortex, hippocampal formation, and septal area [70]. The limbic system is involved in controlling emotion, cognitive functions, and stress responses [70]. Hence, limbic system dysfunctions can lead to pathological conditions, such as dementia [71], epilepsy [72], and anxiety disorders [73]. Glutamatergic neurons are abundant in the hippocampus and amygdala. Interestingly, stimulating those neurons results in an increased free Zn(II) concentration in the synaptic cleft to levels as high as > 100 μM [74]. On the other hand, upregulating synaptic Zn(II) levels can lead to neurotoxicity and promote neurodegenerative diseases, such as Alzheimer’s disease [75]. Since nesfatin-1 immunoreactive neurons were identified in the hippocampus and amygdala nuclei, it seems possible that their involvement in cognitive, stress-response, and depression processes [39] might be Zn(II) dependent. The high levels of Zn(II) ions at the synaptic cleft in vivo could also rationalize the high *K*_d_ value of hN1 observed here in vitro. Moreover, the signals induced and/or modulated by N1 in the amygdala could affect the functions of Nucb1/Nucb2 and NLP/nesfatins in the hypothalamus via the stria terminalis. However, there are no reports on the interactions of the apo- and holo-N1 in the synaptic cleft and its physiological implications that could shed new light on the properties of this unique peptide. Another question is whether N1, N2, and N1/2 display amyloidogenic properties that are more akin to other Zn(II)-dependent amyloid proteins, such as tau [76], amyloid-*β* protein [77], and *α*_2_-macroglobulin [78], or whether they exhibit an inhibitory effect on fibril formation similar to that of Nucb1 [79].

In summary, we demonstrated the Zn(II)-binding ability of nesfatins and described the influence of these ions on the structure of the above peptides. We conclude that in vivo proteolytical processing of the full-length protein could be an activation mechanism that facilitates hN1-cellular trafficking and multiple-partner interactions through the order-to-disorder transition. The observed hN1 oligomerization under Zn(II) might be a prerequisite for interaction with specific ligands and be a part of its complex regulatory framework. We have also demonstrated that the destabilization of hN1/2/hNucb2 in the presence of Zn(II) might arise from the properties of hN2, which presumably also has a structural role. Nonetheless, further studies are necessary to elucidate the role of Zn(II) in nesfatins sensing and its molecular mechanism as well as physiological purpose.

## Supplementary Information

**Additional file 1.** Supplementary figures and tables.

## Abbreviations

ANS: 8-Aniline-1-naphtalene-sulfonic acid
CD: Circular dichroism spectroscopy
ggNucb2: *Gallus gallus* Nucleobindin-2
hN1: Human nesfatin-1
hN1/2: Human nesfatin-1/2
hN2: Human nesfatin-2
hNucb2: Human Nucleobindin-2
IDPs: Intrinsically disordered proteins
IDRs: Intrinsically disordered regions
IMAC: Ion metal affinity chromatography
ITC: Isothermal titration calorimetry
MRE: Mean residue ellipticity
NLP: Nesfatin-1 like peptide
Nucb1: Nucleobindin-1
PC2: Prohormone convertase 2
PC1/3: Prohormone convertase 1/3
PCR: Polymerase chain reaction
SEC: Size exclusion chromatography
SV-AUC: Sedimentation velocity analytical ultracentrifugation
ZF: Zinc finger
ZI: Zincon
