# Supplementary material for: Does one plus one always equal two? Structural differences between nesfatin-1, -2, and nesfatin-1/2
Source: Cell Commun Signal. 2022 Oct 24;20:163. doi: 10.1186/s12964-022-00980-7 (PMC9590162; doi:10.1186/s12964-022-00980-7)
Supplement: Supplementary file 2 — Additional file 1. Supplementary figures and tables. [file 12964_2022_980_MOESM2_ESM.docx]

*Supplementary data*

**Does one plus one equal two? Structural interdependency of nesfatin-1 and nesfatin-2 in the presence of zinc ions**

Rafał Lenda^1^, Michał Padjasek^2^, Artur Krężel^2^, Andrzej Ożyhar^1^, Dominika Bystranowska^1, ✉^

^1^ Department of Biochemistry, Molecular Biology and Biotechnology, Faculty of Chemistry, Wroclaw University of Science and Technology, Wybrzeże Wyspiańskiego 27, 50-370 Wrocław, Poland

^2^ Department of Chemical Biology, Faculty of Biotechnology, University of Wrocław, Joliot-Curie 14a, 50-383, Wrocław, Poland

^✉^ To whom correspondence should be addressed. E-mail: [dominika.bystranowska@pwr.edu.pl](mailto:dominika.bystranowska@pwr.edu.pl)

**Keywords**

Nucleobindin-2, nesfatin-1, nesfatin-2, zinc, intrinsically disordered protein, IDP, metalloprotein, hormone, neuropeptide, satiety molecule

**Figures**


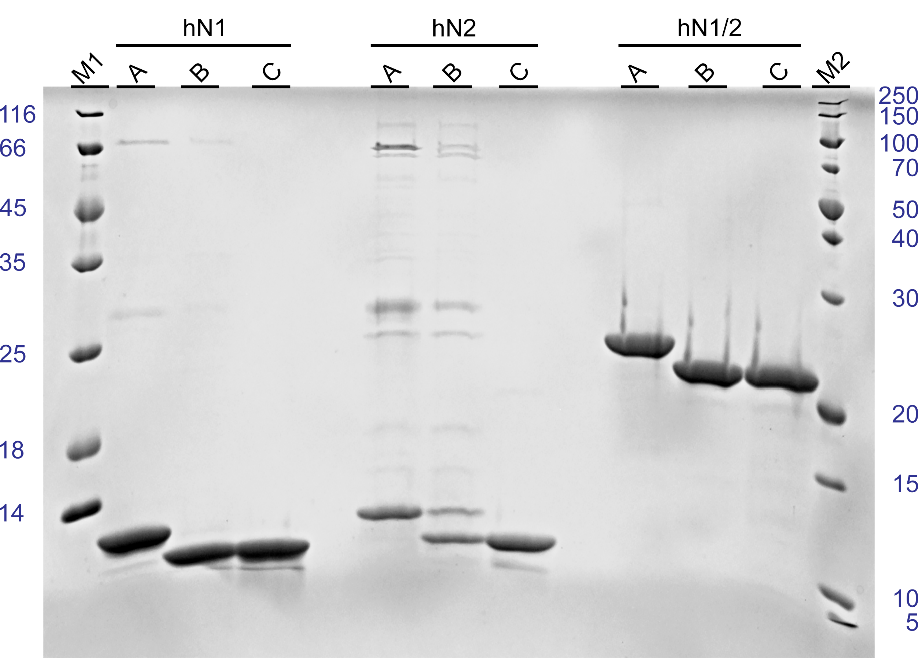


**Fig. S1.** SDS–PAGE analysis of human nesfatins. Lanes M1 and M2 contain protein molecular weight markers. Lane A contains samples pooled after IMAC. Lane B contains samples before SEC. Lane C contains samples pooled after SEC. All lanes were standardized for 5 μg of nesfatin.


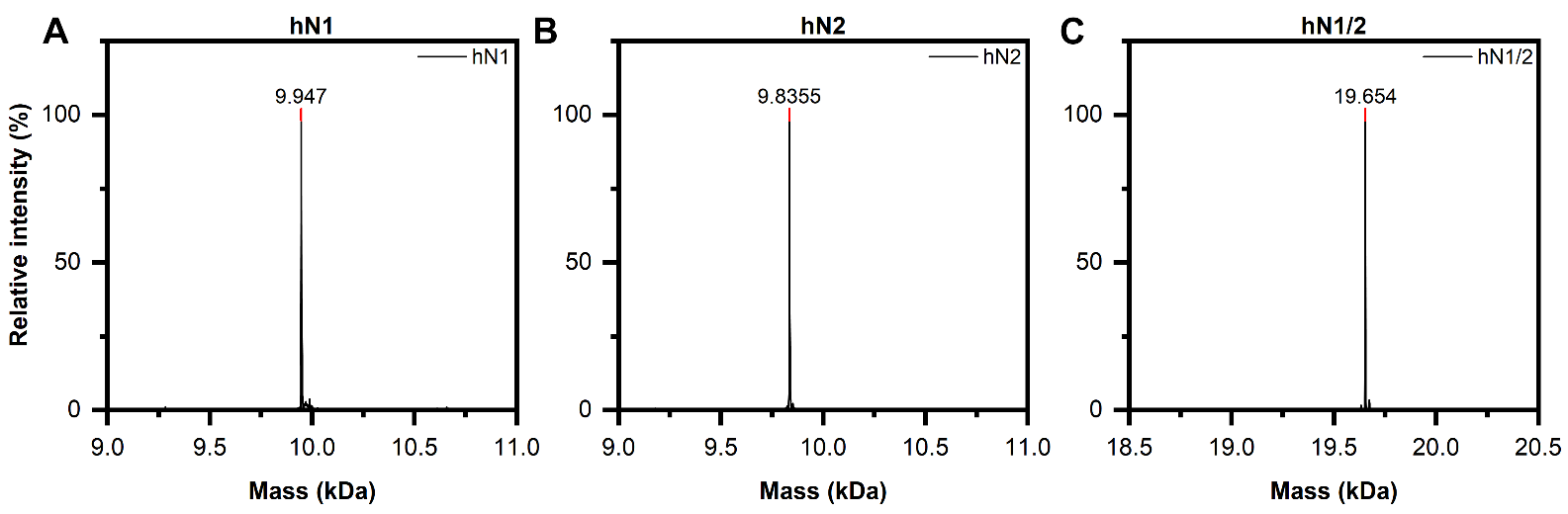


**Fig. S2.** Mass spectra of human nesfatins. (**A**) Mass spectrum of human nesfatin-1, (**B**) Mass spectrum of human nesfatin-2, (**C**) Mass spectrum of human nesfatin-1/2.


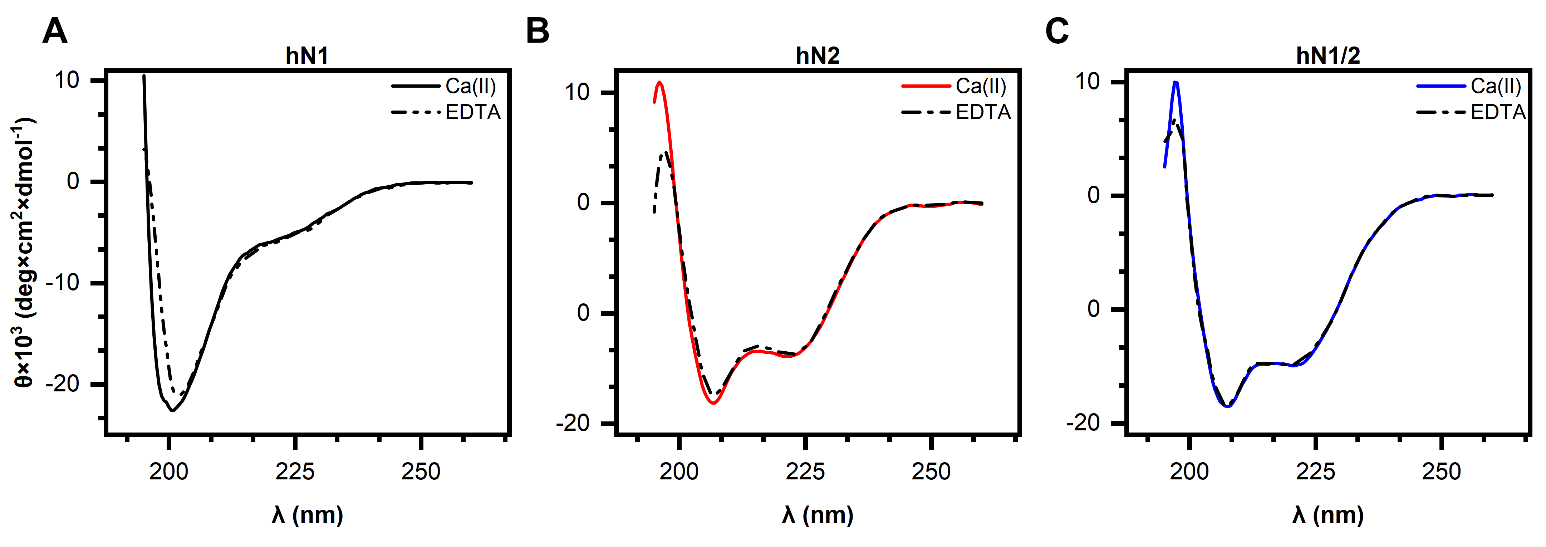


**Fig. S3.** CD spectra of human nesfatins (0.15 mg/ml each) in the presence of 10 mM Ca(II) ions (solid lines) and 5 mM EDTA (dashed lines). CD spectra of (**A**) hN1, (**B**) hN2, and (**C**) hN1/2.


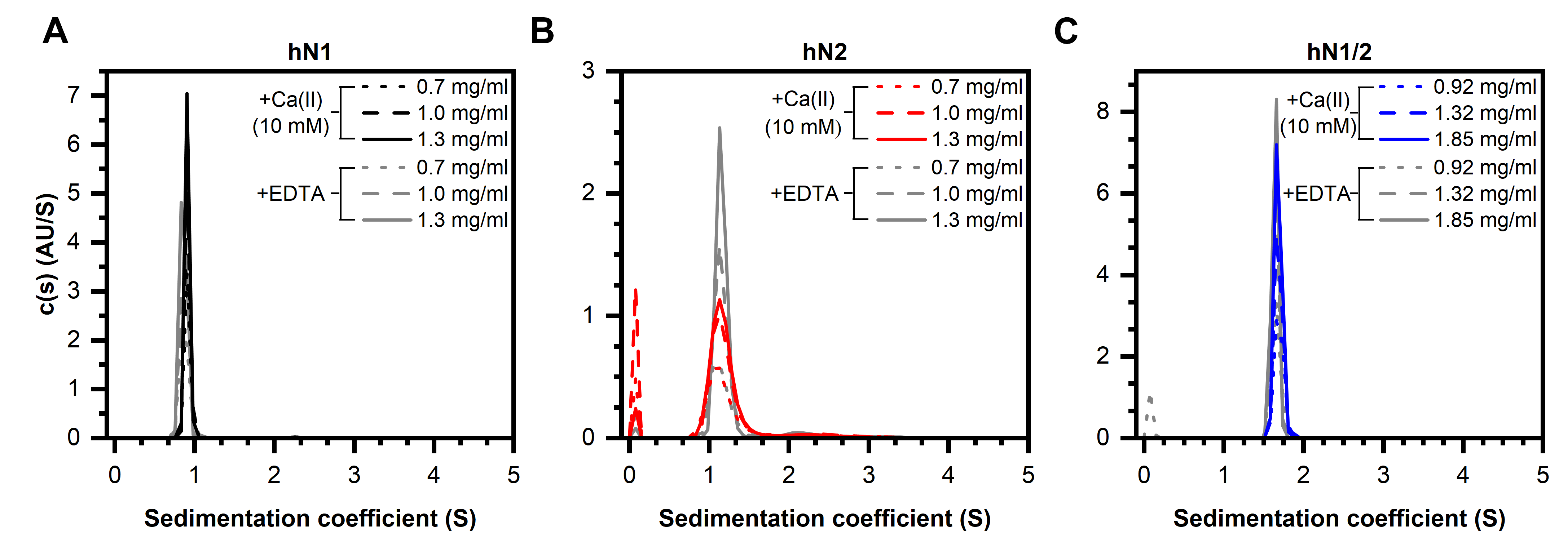


**Fig. S4.** SV-AUC analysis of human nesfatins in the presence of 10 mM Ca(II).


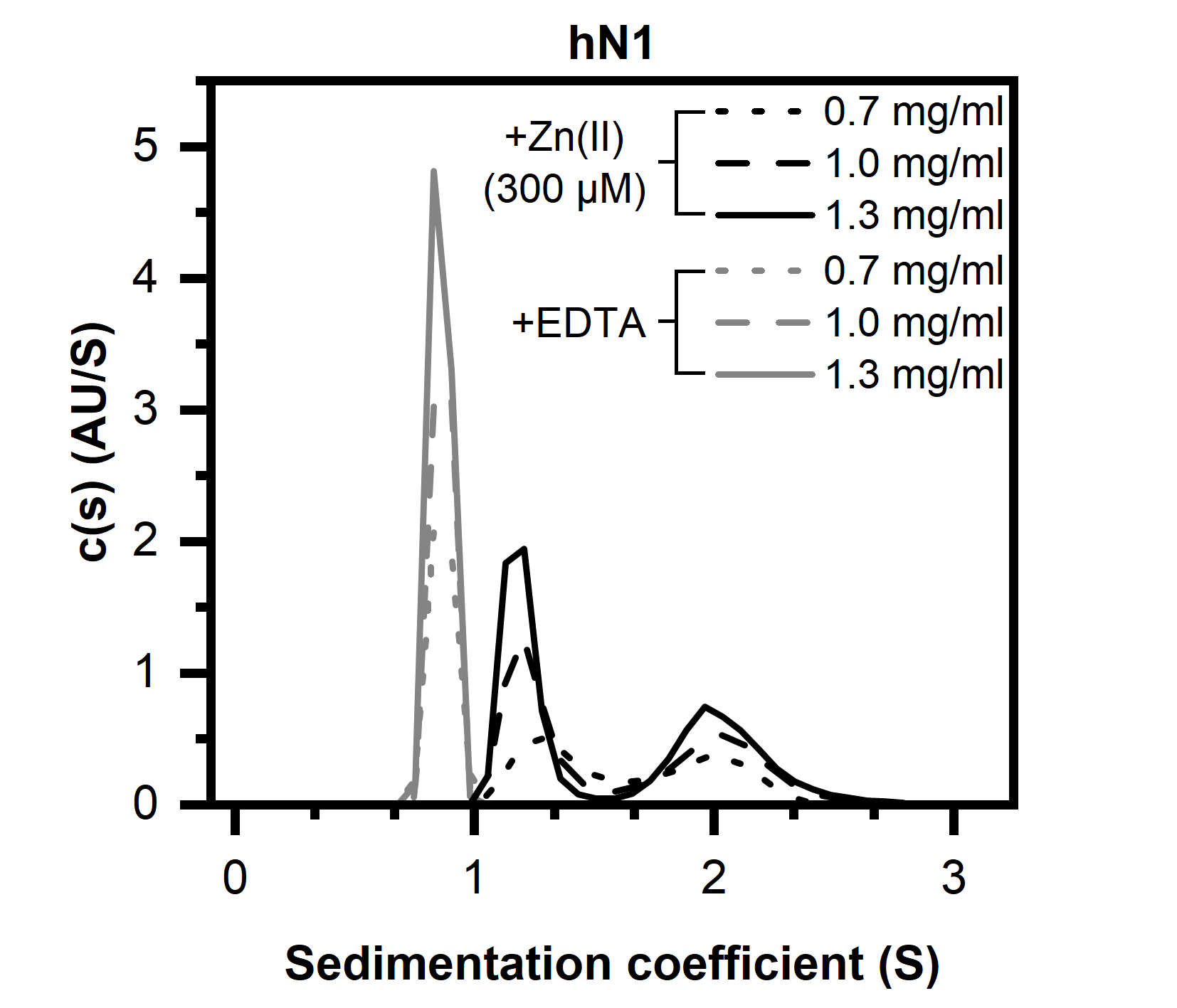


**Fig. S5.** SV-AUC analysis of human nesfatin-1 in the presence of 300 μM Zn(II).


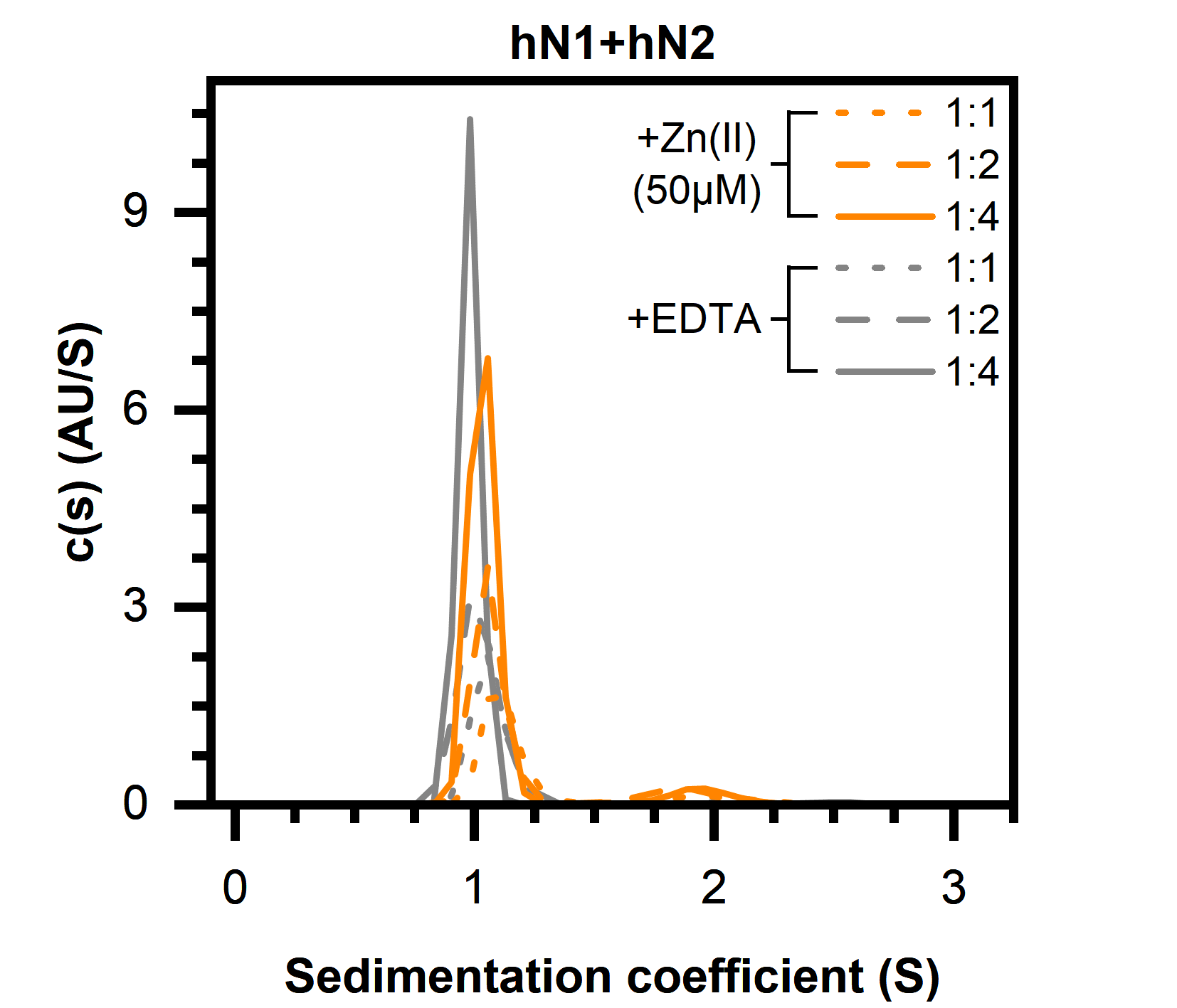


**Fig. S6.** SV-AUC analysis of human nesfatin-1/nesfatin-2 interactions in the presence of 50 μM Zn(II) and 5 mM EDTA. The data were recorded at 1:1, 1:2, and 1:4 w/w ratio (hN2:hN1).


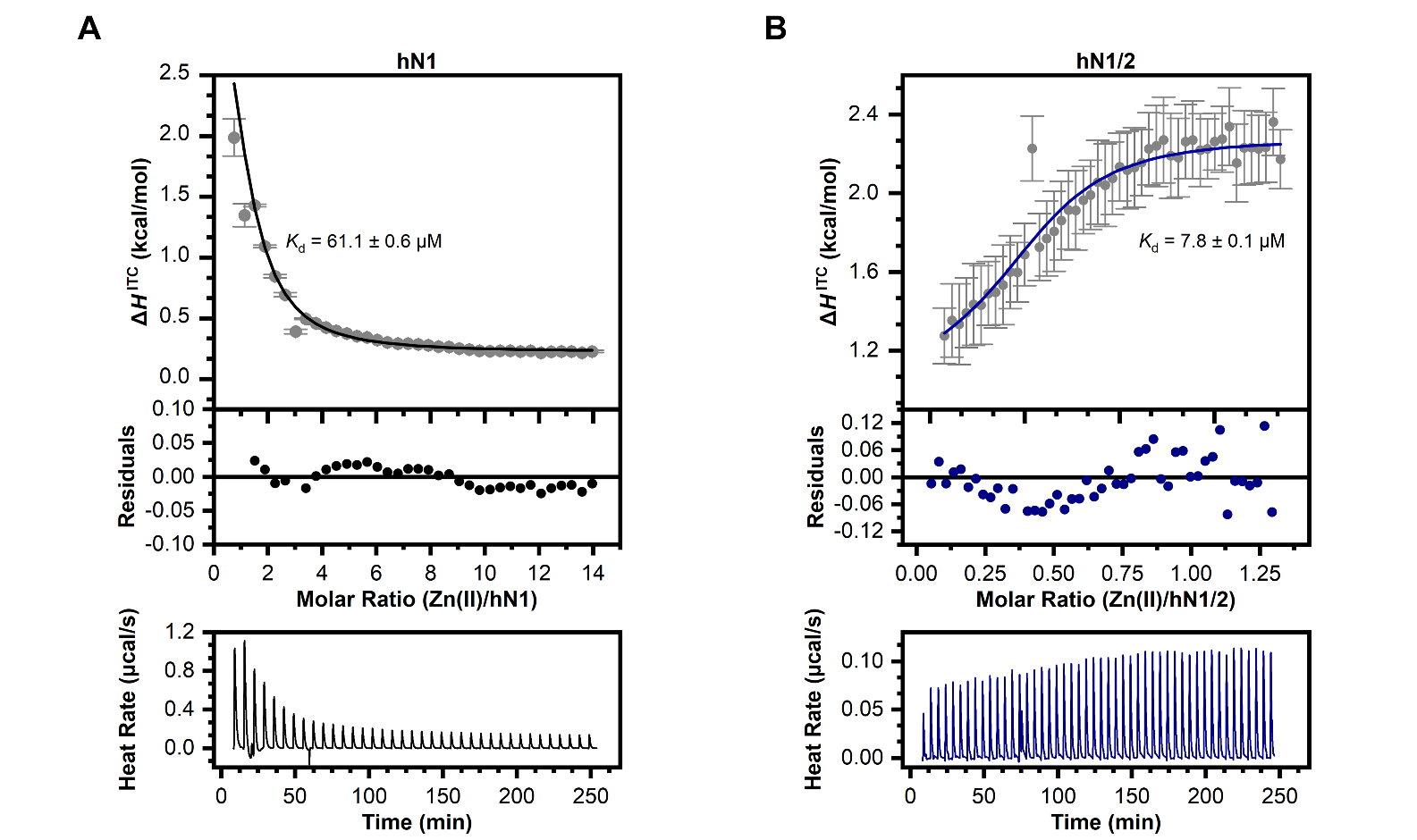


**Fig. S7.** ITC results of human nesfatin-1 and -1/2 titrated with Zn(II) in 20 mM MES, pH 6.5; 150 mM NaCl, represented as functions of heat rates plotted against molar ratios of titrant to titrand (bottom) with corresponding fit (top) and fit residuals (center). (**A**) 0.1 mM hN1 titrated with 7 mM ZnSO_4_. (**B**) 0.1 mM hN1/2 titrated with 0.5 mM ZnSO_4_.


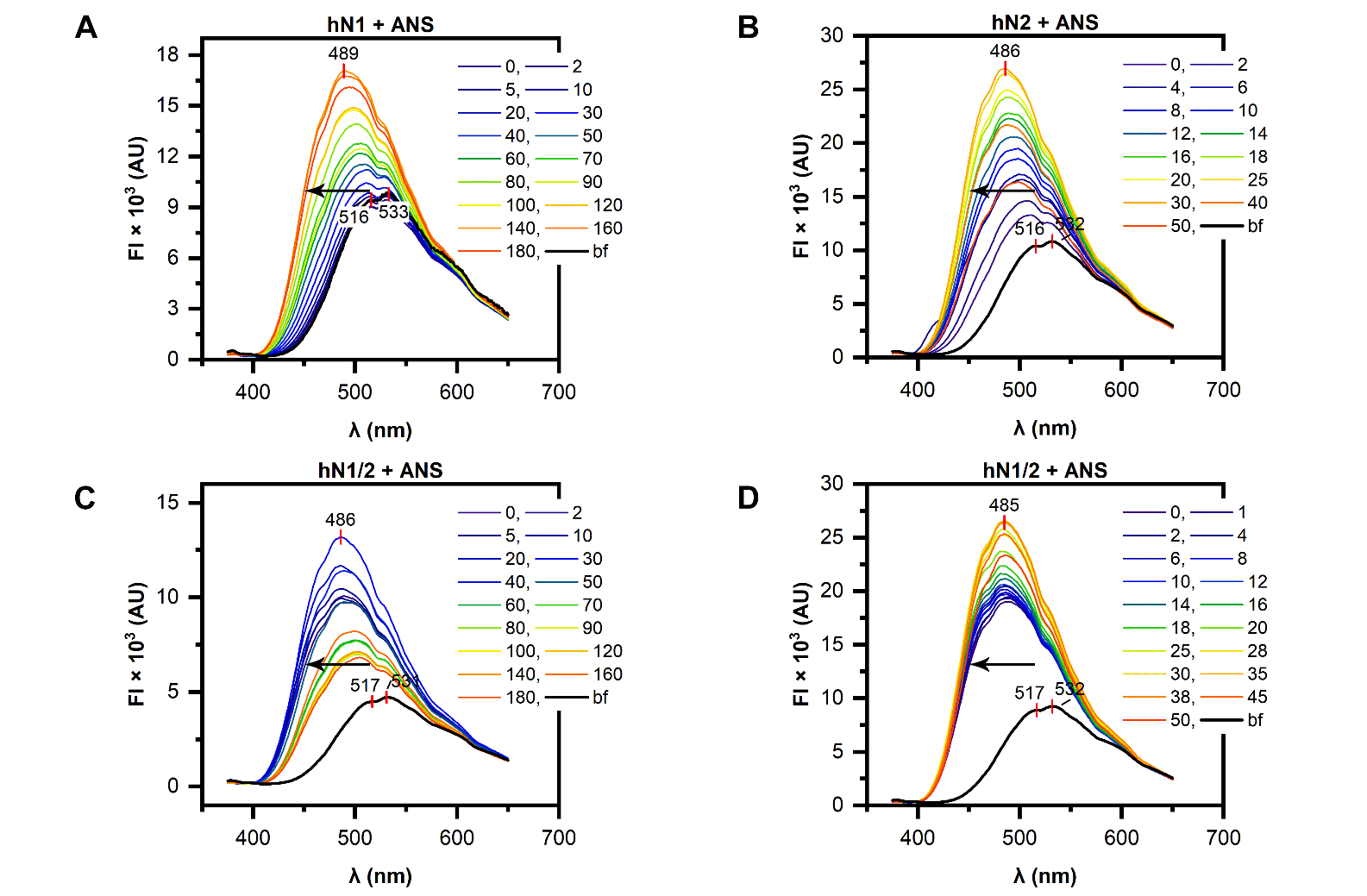


**Fig. S8.** Emission spectra of 50 μM ANS in the presence of Zn(II) and 10 μM: (**A**) nesfatin-1, (**B**) nesfatin-2, (**C**, **D**) nesfatin-1/2. The legend indicates the concentration of Zn(II) ions (μM) and the emission spectra of the free ANS in the buffer (bf).

**Tables**

**Table S1.** Hydrodynamic properties of human nesfatin-1 in the presence of Zn(II) ions.

| Protein | Compound | c [mg/ml] | rmsd | s_(20, w)_ [S] | f/f_0_ | R_h_ [nm] | MW_app_ [kDa] |
| --- | --- | --- | --- | --- | --- | --- | --- |
| hN1 | (300 μM) ZnCl_2_ | 0.7 | 0.006584 | 1.42  2.08 | 1.42 | 2.32  2.80 | 14.5 (54%)  25.9 (46%) |
|  |  | 1.0 | 0.006983 | 1.30  2.14 | 1.34 | 2.02  2.59 | 11.7 (53%)  24.5 (47%) |
|  |  | 1.3 | 0.007537 | 1.25  2.14 | 1.36 | 2.03  2.65 | 11.3 (53%)  25.2 (47%) |
